# Decreasing importance of carbon-climate feedbacks in the Southern Ocean in a warming climate

**DOI:** 10.1126/sciadv.adr3589

**Published:** 2025-05-16

**Authors:** Tereza Jarníková, Corinne Le Quéré, Steven Rumbold, Colin Jones

**Affiliations:** ^1^ University of East Anglia, Norwich, UK.; ^2^ National Centre for Atmospheric Science, University of Reading, UK.; ^3^ National Centre for Atmospheric Science, University of Leeds, UK.

## Abstract

The Southern Ocean is an important CO_2_ sink, mitigating climate change, but its future evolution is uncertain due to the confounding effects of stratospheric ozone recovery and climate change on ocean circulation. Using an Earth System Model, we quantify the relative influence of ozone-depleting substances and greenhouse gas emissions on this sink from 1950 to 2100. Ozone effects dominated changes in ocean circulation during 1950–2000, but not this century, implying that past trends cannot serve as proxies for future changes. Despite substantial future circulation changes induced by climate change, their effect on the CO_2_ sink decreases over the 21st century because of compensating factors. Thus, the Southern Ocean is unlikely to be a major future source of amplifying carbon-climate feedbacks this century.

## Teaser

Over the 21st century, ozone-driven circulation changes to the Southern Ocean carbon sink will be replaced by warming effects.

## INTRODUCTION

Relative to its area, the Southern Ocean accounts for an outsized proportion of the global ocean anthropogenic CO_2_ and heat uptake (*1*–*3*) and regulates atmospheric CO_2_ on century to millennial timescales (*4*). The Southern Ocean is also the largest source of uncertainty in the global ocean carbon cycle, accounting for the largest discrepancies in estimates of the mean ocean carbon sink among carbon cycle models (*5*, *6*), among data products (*6*), and between data products and ocean models (*5*). Research over the past two decades has suggested large decadal variability in the Southern Ocean carbon sink, triggered by variability in ocean circulation, with stagnation in the 1990s (*7*, *8*), a reinvigoration in the 2000s (*9*), and substantial reorganization of carbon in the ocean interior (*10*). Evidence from the Holocene (*11*), from theory (*12*), and from coupled carbon-climate model analysis as part of the IPCC (Intergovernmental Panel on Climate Change) Sixth Assessment Report (*4*) suggests that the Southern Ocean response to a changing climate could generate a positive (amplifying) climate feedback, but its amplitude is uncertain and inter-model spread is large (*2*). Whereas the direct effect of warming of the ocean on the CO_2_ sink is well-known (*13*), the influence of changes in circulation is a dominant source of uncertainty in the assessment of future carbon-climate feedbacks (*4*).

Despite the importance of understanding the response of the Southern Ocean carbon sink to a changing climate, there is no consensus on the processes that have caused past decadal variability, with some studies attributing the changes to strengthening winds from ozone depletion (*7*, *10*), to variability of the Southern Annular Mode (*14*, *15*), or to a complex interplay between dynamic and thermodynamic responses of the natural carbon cycle at the regional level (*9*, *16*, *17*). Understanding and quantifying the role of ozone-depleting substances (ODSs) and greenhouse gases (GHGs) as external climatic drivers and the response of the oceanic carbon cycle to the changes in ocean properties that they induce is fundamental to understanding and projecting the strength and evolution of the Southern Ocean carbon sink this century and beyond and to constraining future carbon cycle–climate feedbacks.

Depletion of stratospheric ozone and the accumulation of GHGs in the atmosphere have both been demonstrated to exert an influence on ocean dynamics in past decades. Depletion of stratospheric ozone in the latter half of the 20th century has driven an acceleration and poleward shift of Southern Ocean winds, particularly in the austral summer (*18*). Southern Hemisphere ozone concentrations reached a minimum around 1990 and have slowly been recovering since due to the adoption of the 1987 Montréal Protocol (*19*), which led to a phasing out of ODSs (*20*). Simultaneously, an increase in GHGs has led to a warming and freshening of the Southern Ocean and an associated stratification of the surface layer (*21*). Models suggest that the increase in GHGs also induced a strengthening of the winds year-round (*22*–*24*), which augments the intensification of winds due to ozone loss. In the Southern Ocean, the upper meridional overturning circulation (MOC) is broadly expected to increase in response to wind intensification (*25*–*27*), but the strength of this response is disputed, depending on the relative strength of eddy saturation (*28*), and is sensitive to changes in surface buoyancy forcing (*29*, *30*).

The expected recovery of stratospheric ozone will combine with the continued rise in GHG concentrations this century, potentially influencing physical ocean properties and the ocean carbon sink in multiple interacting ways. Predicting the future evolution of the Southern Ocean wind fields is central to identifying and quantifying their effects on the physical ocean sea state and the carbon sink. The combined effects of physical drivers of the ocean carbon sink can oppose each other in complex ways. Stronger winds deepen the mixed-layer depth (MLD), ventilating carbon-rich waters or, conversely, bringing nutrients that may stimulate primary productivity and biological drawdown. An increase in the MOC strength is primarily likely to enhance the ventilation of deeper natural carbon-rich waters to the surface, although it may simultaneously stimulate deep water formation and, thus, anthropogenic carbon drawdown (*31*, *32*). Warming of sea surface temperatures (SSTs) in response to increasing GHG concentrations lowers the solubility of CO_2_ and decreases the carbon sink efficiency (*13*). The response of SSTs to wind strengthening due to ozone loss can also vary in time, with an initial surface cooling due to northward Ekman transport giving way to warming resulting from enhanced upwelling of warmer waters due to a stronger MOC (*33*).

The net effect of changing ozone and GHG concentrations on the evolution of the Southern Ocean carbon sink thus depends on the relative magnitudes of the different physical drivers and will combine with the uptake of CO_2_ by the ocean in direct response to changing atmospheric CO_2_ concentration. The effect of climate change and variability on the Southern Ocean carbon sink has been shown to be as large as the direct response to increasing atmospheric CO_2_ concentration in recent decades (*10*). Here, we quantify these relative influences over the Southern Ocean, defined here as the region south of 50°S, using the UK Earth System Model (UKESM1) (*34*) in combination with a range of biogeochemical observations.

We conduct six simulations designed to separate the effects of ODSs and GHGs over the time period 1950–2100. We design three scenarios of prescribed ODS that can be thought of as no ozone loss, ozone loss and recovery, and ozone loss with no recovery (see Materials and Methods for a full description of how ozone evolution is simulated as a result of the prescribed ODS scenarios). We combine each ODS scenario with a high– and a low–GHG emission scenario under two shared socioeconomic pathways [SSP 3-7.0 and SSP 1-2.6; (*35*)]. We isolate the effects of ozone and GHG on winds and on oceanic properties of SST, MLD, and MOC, focusing on three 50-year intervals corresponding to a period of maximum ozone loss (1950–2000), mixed ozone/GHG effects (2000–2050), and maximum GHG effect (2050–2100; see Materials and Methods). We then infer their relative influence on the evolution of the Southern Ocean surface carbon concentration and carbon-climate feedback, using both the biogeochemical model output of UKESM1 (*36*) and biogeochemical values constrained by available observations (see Materials and Methods).

## RESULTS

### Changing wind patterns and dominant drivers

**Fig. 1.**
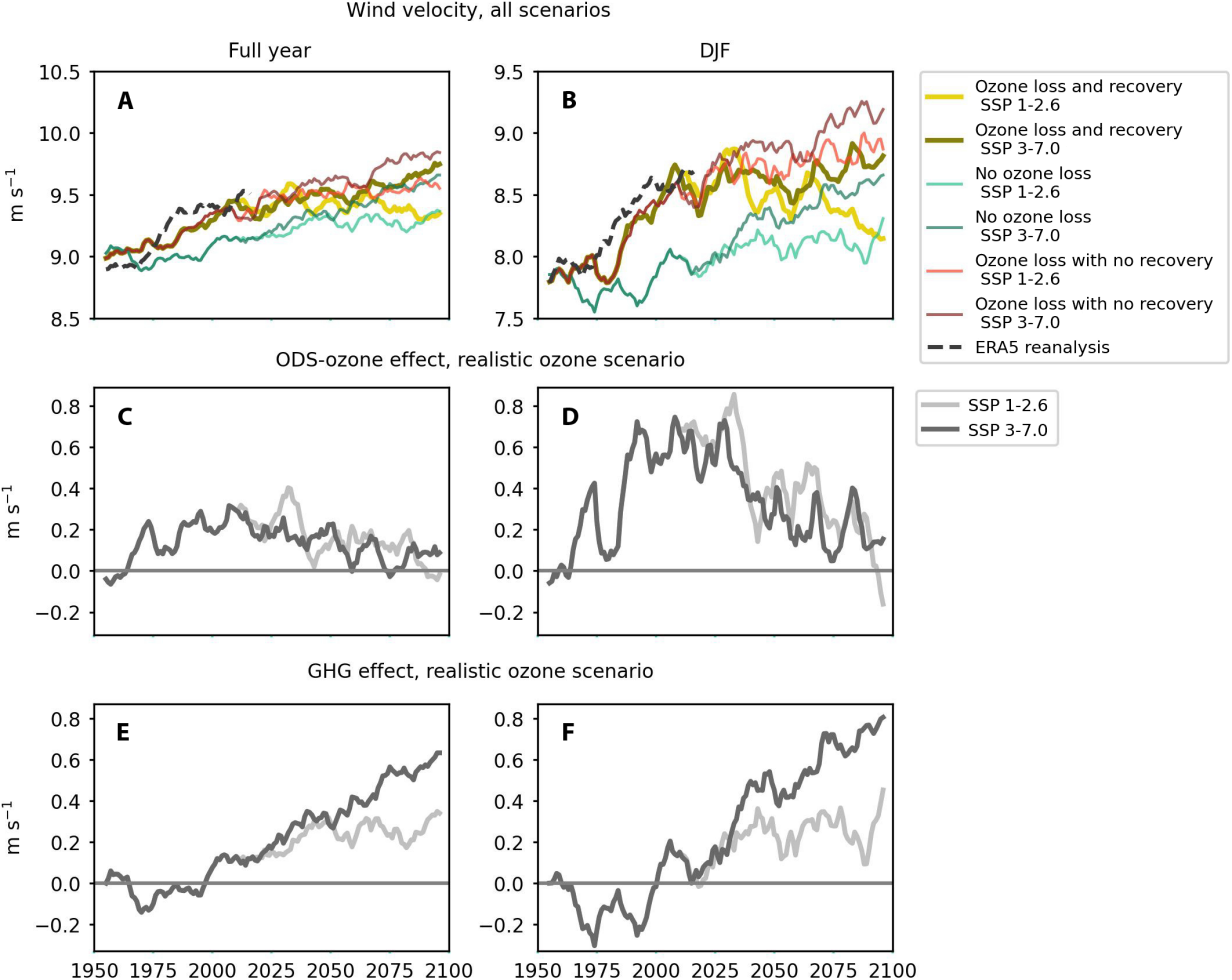
Southern Ocean mean open-water wind speed, 1950–2100 (in meters per second). ERA5 (European Centre for Medium-Range Weather Forecasts Reanalysis v5) reanalysis shown until 2020 for comparison. Year-round (left) and austral summer [December-January-February (DJF)] (right) mean wind speed for all six scenarios conducted with the UK Earth System Model (UKESM1) (**A** and **B**), as well as a decomposition of the contribution of ODS changes (which imply ozone changes) (**C** and **D**) and GHG changes (**E** and **F**) to wind speed trends under the realistic ODS scenario (with ozone loss to 1990 and recovery thereafter) and the two shared socioeconomic pathway (SSP) scenarios (SSP 1-2.6 and SSP 3-7.0). See the “Estimation of effect of ozone and GHG trends on changes in wind and ocean fields” section for the method used to decompose the signal into the two factors. The Southern Ocean region is taken as south of 50°S in this analysis, and time series are smoothed with a 10-year running mean. (See fig. S1 for a version of this figure showing the *u* component of the winds between 40°S and 60°S.)

South of 50°S, winds increase between 1950 and the end of the 21st century in all seasons, with the strongest trend in the second half of the 1950–2000 period in scenarios with ozone loss (Fig. 1, A and B; see fig. S1 for the behavior of the westerlies averaged between 40°S and 60°S, which is qualitatively similar). Ozone depletion is dominantly responsible for the observed changes during this historical period, while the effect of GHGs is not statistically significant. Ozone-induced wind acceleration is strongest in austral summer [December-January-February (DJF); 0.12 m s^−1^], followed by austral spring [September-October-November (SON); 0.09 m s^−1^], with no statistically significant trend over 1950–2000 in other seasons (see table S1 for all trends).

Wind trends shift from ozone-dominated to GHG-dominated during the 21st century. The GHG contribution to wind increase becomes visible at the beginning of the 21st century and ramps up steadily through the century, with the end-of-century total GHG effect size over the time period under SSP 3-7.0 (0.63 m s^−1^) nearly double that under SSP 1-2.6 (0.35 m s^−1^). Unlike the ozone effect, the GHG effect is of similar magnitude in all seasons and, except in austral summer, is stronger under the SSP 3-7.0 scenario than the effect of ozone loss.

The recovery of ozone loss visible from around 2030 contributes to wind deceleration, while the effect of increasing GHGs on wind acceleration depends on the emissions magnitude, with wind speeds increasing more in the higher GHG scenario in all three ozone scenarios. The final trend in wind speed, therefore, depends on which forcing dominates. In the low–GHG emission scenario, winds slow down beginning in mid-21st century, reaching an end-of-century (2090–2100) value that is only 0.35 m s^−1^ above average wind magnitudes in 1950–1960. In contrast, in the high–GHG emission scenario, wind speeds first level off as the effect of ozone recovery offsets the effect of GHGs but then increase again, reaching an end-of-century value of 0.75 m s^−1^ above wind magnitudes in 1950–1960, showing that the effects of external climatic drivers on the Southern Ocean wind structure are partially reversible, but only under a low GHG scenario. Analogous effects of ozone and GHGs are seen in the latitudinal movement of the maximum wind speed (the wind jet) (fig. S2), with GHG increases and ozone depletion both acting additively to push the jet poleward and ozone recovery under a low-GHG scenario leading to an equatorward retreat of the wind jet to near its initial position.

### Shifting controls of external climate forcings on physical ocean properties

**Fig. 2.**
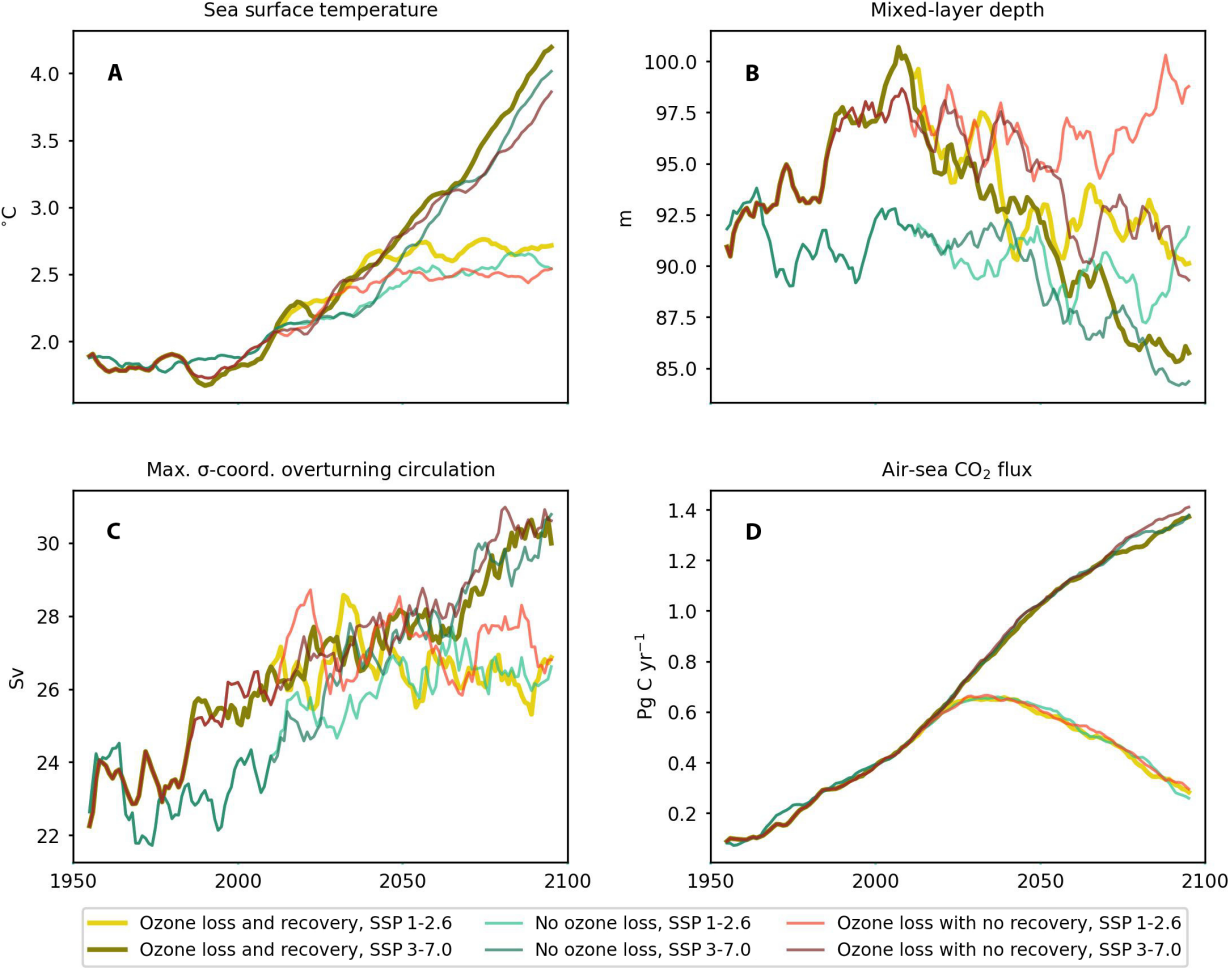
Annual mean physical variables most influencing the ocean carbon cycle and air-sea CO_2_ flux, 1950–2100. Yearly average time series are for SST (°C) (**A**), MLD [meters (m)] (**B**), maximum σ-coordinates MOC [Sverdrup (Sv)] (**C**), and air-sea CO_2_ flux [petagrams of C year^−1^ (Pg C yr^−1^)] (**D**). For MOC, the value given is the maximum of the yearly average overturning at or below 50°S, whereas other variables are averaged poleward of 50°S. Seasonally subdivided time series are shown in fig. S3. Time series are smoothed with a 10-year running mean.

The evolution of SSTs primarily follows each GHG scenario and is a response to the associated radiative forcing (Fig. 2A). Warming in the SSP3-7.0 scenario is approximately double that in SSP 1-2.6 over the entire period 1950–2100 (Fig. 2A). Despite the leading-order control of the GHG scenario on SST, a minor, opposing, ozone effect is visible, especially in austral summer (fig. S3B), with an initial surface cooling due to ozone depletion visible during 1980–2000. The GHG-induced SST warming is likely high due to UKESM1 having a high equilibrium climate sensitivity (*37*).

The fate of the MLD is determined by a balance between thermal and freshwater-driven shoaling and wind-driven mixing. In the second half of the 20th century, the MLD deepens primarily because of ozone depletion–driven wind intensification and associated mixing that outcompetes the GHG-driven thermal shoaling (Fig. 2B), before subsequently shoaling throughout the 21st century, more strongly in SSP 3-7.0 than SSP 1-2.6, suggesting the GHG-associated thermal shoaling outcompetes the increased wind-mixing under the high-GHG scenario. This pattern supports the mechanism suggested by Sallée *et al.* (*21*), who proposed that the observed past deepening of the MLD may be caused by a strengthening wind field counteracting and overpowering the expected warming effects. Strong GHG-driven shoaling is seen in the high-GHG scenarios in austral winter and spring, but not in summer (fig. S3, G to J), suggesting that similar levels of warming have a stronger absolute impact on the MLD in the less-stratified winter water column. A contrasting response is seen in the maximum MOC, which is strengthened both by ozone depletion and GHG forcing, following the changes in wind velocity (Fig. 2C). An apparent shift in controls on overturning is seen from the end of the 20th century, when the scenarios are divided along ozone-effect lines, to the end of the 21st century, when they are divided by SSP scenario (Fig. 2C).

**Fig. 3.**
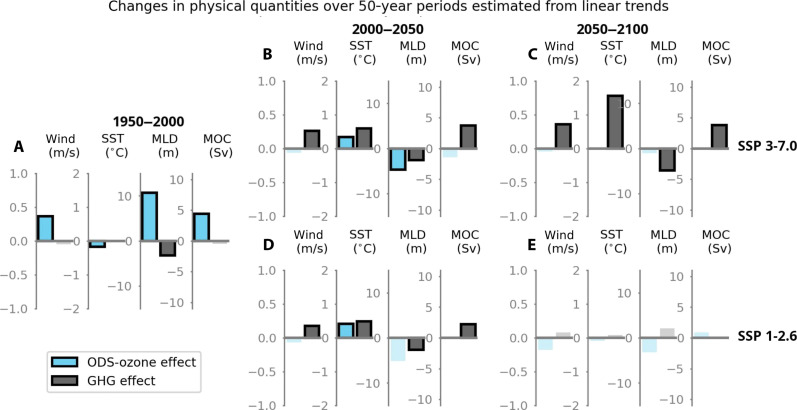
Contribution of ODS (blue bars) and GHG emissions (gray bars) to 50-year changes in mean open-water wind speed, SST, MLD, and MOC estimated from linear trends. (**A**) Historical time period 1950–2000; (**B**) 2000–2050, SSP 3-7.0; (**C**) 2050–2100, SSP 3-7.0; (**D**) 2000–2050, SSP 1-2.6; (**E**) 2050–2100, SSP 1-2.6. Nonsignificant trends are grayed out.

Separating the cumulative ozone and GHG effects in each of the three 50-year subperiods of the time series makes clear the general shift in controls that takes place in the second half of the 21st century (Fig. 3). During 1950–2000 substantial and significant ozone-related trends are seen in all physical fields (Fig. 3A), which begin to diminish in the first half of the 21st century as GHG-forced changes become more apparent. In the second half of the 21st century, ozone-related changes are no longer significant, while GHG-forced changes bifurcate on SSP scenario, with large significant trends in all fields in SSP 3-7.0 (Fig. 3C) and no significant trends in SSP 1-2.6 (Fig. 3E). The impact of ozone depletion on the physical characteristics of the Southern Ocean is gradually superseded by the increasing impact of GHG-forced climate change.

### Implications for the Southern Ocean carbon sink and carbon-climate feedbacks

The Southern Ocean CO_2_ sink grows continuously in the high-emission scenarios but decreases toward its 1980 value by 2100 in the low-emission scenarios (Fig. 2D), as seen in other earth system models (ESMs). This dominant signal represents the direct response of the ocean carbon cycle to changes in atmospheric CO_2_ concentration and is primarily controlled by well-known carbonate chemistry and the mean vertical transport of water between the surface and the deeper ocean (*38*). The response of the ocean carbon cycle to changes in physical properties driven by ozone and GHGs, which is a potential source of amplifying carbon-climate feedbacks, is estimated here using well-known relationships of solubility (*13*) and dynamical processes (*39*) on surface dissolved inorganic carbon (DIC). The response of surface DIC to changes in physical transport is directly dependent on the vertical profile of DIC, which is weaker than observed in UKESM1 (see fig. S4 and Materials and Methods). To circumvent this common model bias (*40*), we estimate the specific contribution of ozone and GHG on DIC in two ways: first, using biogeochemical values constrained by observations, and, second, using biogeochemical values obtained directly from the UKESM1 ocean biogeochemical component (see Materials and Methods).

**Fig. 4.**
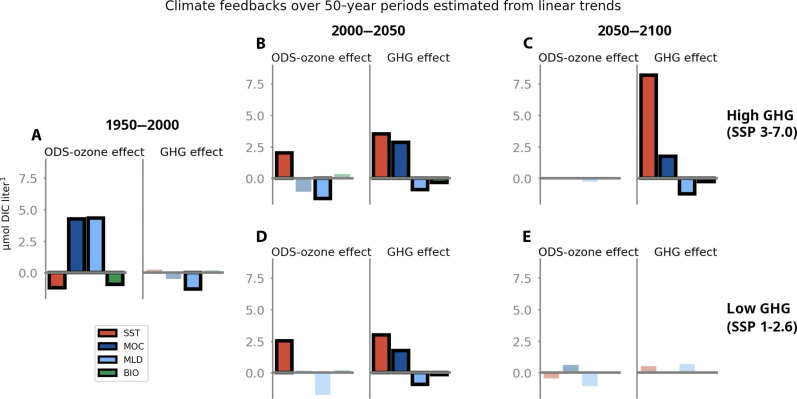
Effective contribution of ozone-forced and GHG-forced climate feedbacks from SST, MOC, MLD, and biological carbon drawdown (BIO) to mean changes in surface Southern Ocean DIC concentrations (south of 50°S), using observationally constrained biogeochemical values. DIC changes due to temperature changes are calculated as the change in DIC storage capacity at air-sea equilibrium for a given initial surface pCO_2_ in response to a change in temperature (see Materials and Methods). Feedbacks estimated from statistically significant linear trends are outlined in bold black. (**A**) Historical time period 1950–2000; (**B**) 2000–2050, SSP 3-7.0; (**C**) 2050–2100, SSP 3-7.0; (**D**) 2000–2050, SSP 1-2.6; (**E**) 2050–2100, SSP 1-2.6. For a version of this figure with model-derived biogeochemical values, see fig. S5.

In the historical 1950–2000 period, ozone-driven deepening of the MLD and enhanced MOC contribute to a combined increase in surface DIC of order 8 μM based on the observationally constrained estimate (Fig. 4A), encouraging a sink reduction compared to the scenario without ozone depletion, helping to explain the observed stagnation of the Southern Ocean carbon sink in the 1990s (*7*, *9*). This DIC increase is partly opposed by an ozone-driven surface cooling that leads to an order ~1 μM increase in equilibrium DIC capacity. Simultaneously, ozone-enhanced surface iron delivery stimulates biological productivity that leads to a ~1 μM decrease in surface DIC (Fig. 4A). Over this period, GHG-driven effects on the carbon cycle are small and limited to a MLD shoaling–driven decrease in DIC of ~1 μM (Fig. 4A), for an overall surface DIC increase of 5 μM when the separate effects of all GHG and ozone-driven physical changes are summed. The total overall surface DIC increase over this period based on UKESM1 biogeochemical values is slightly over 3 μM (see fig. S5), with a reduced effect of MLD and a negligible ecosystem response, due to the reduced vertical gradients in the model compared to observations (see Materials and Methods). In this period, ozone-driven effects dominate over GHG-driven ones in both the model-derived estimate and the observationally constrained estimate.

During the first half of the 21st century, ozone effects reverse as ozone levels begin to recover, slowing down winds. MLD shoaling following ozone recovery decreases surface DIC, an effect that is counteracted by that of ozone recovery–driven warming decreasing the equilibrium DIC capacity (Fig. 4, B and D). Simultaneously, GHG-driven surface warming and associated reduction in DIC capacity becomes more prominent, accompanied by a slight increase in surface DIC due to enhanced overturning that is partly compensated by the opposite effect of a shoaling MLD and a small decrease in biological productivity. These effects are qualitatively similar in both methods, but the roles of MOC and MLD are smaller in amplitude when using model biogeochemical values (fig. S5, B and D).

**Fig. 5.**
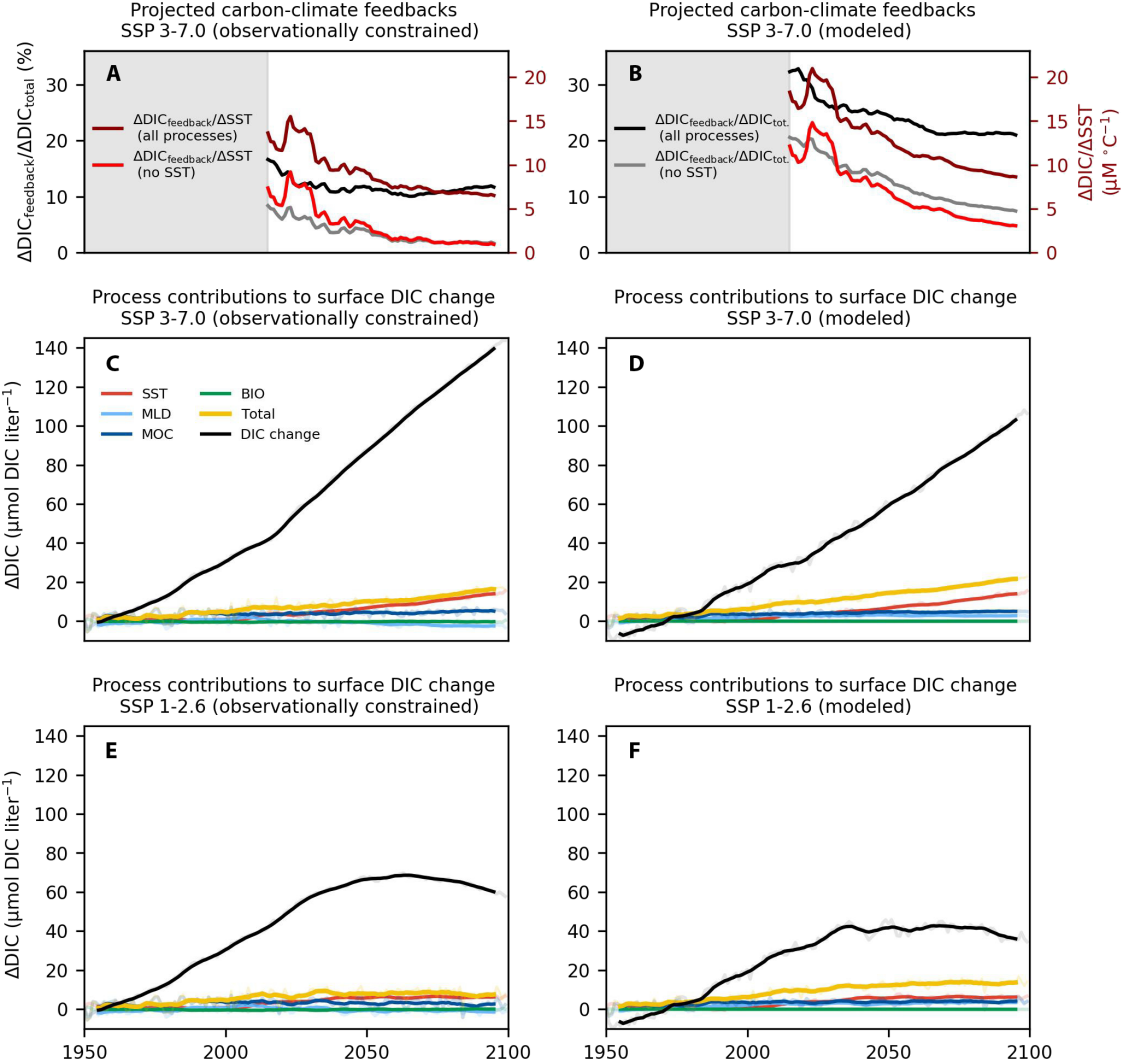
Process contributions to surface DIC change and associated carbon-climate feedbacks using observationally constrained (left column) and model simulated (right column) biogeochemical values. (**A** and **B**) Projected cumulative carbon-climate feedbacks in the Southern Ocean under a high-emission scenario (SSP3-7.0). Left axis: black line represents (cumulative process–driven changes in DIC, all processes)/(total change in DIC), while gray line represents (as above excluding SST effects)/(total change in DIC); right axis: the maroon line shows (cumulative process–driven changes in DIC, all processes)/(cumulative change in SST), while the red line shows (as above excluding SST effects)/(cumulative change in SST). (**C** to **F**) Cumulative changes in surface DIC concentrations broken down into the effective cumulative contribution of changes in individual processes in the Southern Ocean (in micromolar) under high (SSP 3-7.0) (C and D) and low (SSP 1-2.6) (E and F) emission scenarios. The left column uses observationally constrained values for the process contributions and the total change in DIC, while the right column uses modeled values for both (see Materials and Methods and fig. S6). Time series are smoothed with a 10-year running mean.

During the second half of the 21st century, ozone-related effects on physical properties and surface DIC are negligible and not statistically significant. GHG-driven effects on physical properties remain large in the high-emission scenario during 2050–2100, with an approximate warming of 1.6°C, mixed-layer shoaling of 5.9 m, and an increased strength of MOC by 3.6 sverdrup estimated from linear trends (Fig. 3C). However, only the SST warming translates to a substantial effect on the ocean carbon cycle, with an estimated reduction in DIC capacity of ~8 μM (Fig. 4C). While GHG-forced MLD shoaling and MOC increases are large and significant during this period (Fig. 3C), they do not substantially change the surface DIC balance, because the DIC gradient with depth is substantially weakened due to the additional anthropogenic DIC in the surface ocean. Furthermore, the continued shoaling of MLD and increasing MOC have small opposite effects on surface DIC that nearly cancel (Fig. 4), leaving only the effect of SST. The overall feedbacks translate to an estimated increase in DIC of 8 μM in the high-emission scenario, when calculated either with the observationally constrained biogeochemical values or with model values (Fig. 4C and fig. S5). These carbon-climate feedbacks are substantial but limited in amplitude compared to the total model surface DIC change of 47 μM between 2050 and 2100 in the high-emission scenario (Fig. 5D). In contrast, the GHG-related effects on physical properties and surface DIC are negligible in the low-emission scenario. These compensating effects explain why, although substantial changes in SST, MLD, and MOC are projected, with clear disparities across the six scenarios, the combined effects on the Southern Ocean CO_2_ sink are barely distinguishable among scenarios and appear predominantly driven by atmospheric CO_2_ (Fig. 2D).

The carbon-climate feedbacks on surface DIC concentration show a distinct regime shift from primarily ozone-forced, circulation-driven DIC effects in the second half of the 20th century to entirely GHG-forced and primarily temperature/solubility-driven effects in the second half of the 21st century under a high-GHG scenario (Fig. 4, A versus C). Only the SST-driven reduction in surface equilibrium DIC capacity increases in absolute terms over the 21st century (Fig. 5, C and D, red line), while cumulative circulation-driven impacts dominate the total signal at the beginning of the historical period and then stagnate (Fig. 4, A versus C; Fig. 5, C and D). Furthermore, under the high-emission scenario, the ratio of total carbon-climate feedbacks [yellow line in Fig. 5 (C and D), summing individual contributions of all processes] to the total surface DIC change [black line in Fig. 5 (C and D)] declines in the future projection, with the sink magnitude driven primarily by changing atmospheric CO_2_ [ratio shown as black line in Fig. 5 (A and B)]. The decline of the ratio is even steeper when only the dynamical effects are considered, excluding warming effects on solubility [i.e., the sum of all processes except SST change, gray line in Fig. 5 (A and B)]. Likewise, under a different measure, the ratio of carbon-climate feedbacks to surface warming declines over the course of the 21st century, as the carbon-climate feedbacks grow more slowly than the warming itself (Fig. 5, A and B; here, the dark red line represents the rate of change of DIC due to all processes per degree of warming, and the bright red line represents the same excluding SST).

## DISCUSSION

Our analysis suggests that the carbon-climate feedback in the Southern Ocean is decreasing this century. Despite this emerging finding, quantifying the exact amplitude of the ocean carbon-climate feedback remains difficult. The use of one single model (UKESM1) for simulating stratospheric ozone and GHG interactively means that their relative importance as drivers of wind changes could be model dependent. Likewise, the relative response of the MLD and overturning to forcing conditions will also be model dependent. We showed here that the combined effects on temperature will increasingly dominate the response of the ocean carbon cycle to changing conditions this century. Analysis with several ESMs will be needed to gain confidence on the exact evolution of physical conditions and surface temperature in the Southern Ocean this century. Any future changes will also naturally depend on future emissions of both ODS and GHG.

Furthermore, our analysis cannot entirely rule out the possibility that additional carbon-climate feedbacks, mediated by changes in marine ecosystems, take place, beyond the direct effects of changes in nutrients resulting from changes in physical dynamics. Ecosystem-climate feedbacks have been highlighted as a “known-unknown” in the last four assessment reports of the IPCC, with only slow progress in their incorporation into models and in the development of observational constraints to determine their sign or size (*41*, *42*). Ecosystem-mediated feedbacks have taken place in the geological past (*43*) and could influence the stabilization level of atmospheric CO_2_ in the long term.

Nevertheless, the results presented here demonstrate several important factors that constrain the evolution of the Southern Ocean CO_2_ sink. First, trends over recent decades (at least from 1970 to present) cannot be used to project and even constrain the response of the Southern Ocean to future climate change because of the shifting controls on ocean physical properties from ozone-dominated trends in the past decades to GHG-dominated trends in the 21st century. Second, wind-driven changes forced by ozone depletion should reverse substantially this century, including their effect on physical properties of the Southern Ocean. Third, even if there are large future changes in overturning and MLD, their influence on the ocean CO_2_ sink will decrease with time because of the reduction in the DIC vertical gradient driven by the uptake of anthropogenic carbon and because the effects of GHG-driven MLD shoaling and enhanced overturning on surface DIC compensate each other.

Because of these evolving and competing factors, in the high-emission scenarios, the carbon-climate feedbacks become almost entirely dominated by the effect of surface warming on CO_2_ solubility, which is well represented in all ESMs used for climate projections. The trend of declining carbon-climate feedbacks over the 21st century, which becomes dominated by well-constrained warming effects, is clear both when estimating it with biogeochemical values derived from UKESM1 and with values constrained by observations [Fig. 5, left column (observations) versus right column (model)] and is, therefore, robust to uncertainty in the biogeochemical fields (see fig. S6 for an additional sensitivity estimate). Our analysis, therefore, indicates that the Southern Ocean is unlikely to become a major source of amplifying carbon-climate feedbacks this century, despite being the origin of the largest source of uncertainty and spread in model projections. Based on the results presented here, efforts to reduce model spread and constrain projections of the ocean carbon cycle in the future should focus on improving the representation of mean ocean transport in models, including mode water formation (*17*), eddy-induced transport (*44*), MOC, and deep-water formation at the ice edge.

## MATERIALS AND METHODS

### UKESM1 model description

We use UKESM1, a well-established ESM containing the global coupled atmosphere-ocean climate model, HadGEM3-GC3 (*34*). We refer to (*34*) for a general evaluation of UKESM1 and only highlight components of the model that are central to our work. The atmospheric component of UKESM1 is the Global Atmosphere 7.1 science configuration of the Unified Model (*45*), with horizontal resolution of ~135 km (1.25° × 1.875°) and 85 vertical levels. The UKESM1 ocean model is the global ocean general circulation model Nucleus for European Modelling of the Ocean version 3.6, configured on a 1° tripolar grid with 75 vertical levels and an explicit nonlinear free surface (*46*). The ocean carbon cycle in the UKESM1 (Fig. 4D) is represented using the MEDUSA (Model of Ecosystem Dynamics, Nutrient Utilisation, Sequestration and Acidification) ocean biogeochemistry model, an intermediate-complexity plankton ecosystem model with a dual size-structured ecosystem of small (nanophytoplankton and microzooplankton) and large (microphytoplankton and mesozooplankton) components; all large phytoplankton are treated as diatoms and require silicic acid for growth (*47*). MEDUSA explicitly resolves the biogeochemical cycles of carbon, alkalinity, and dissolved oxygen, as well as of nitrogen and silicon, and includes an implicit representation of iron.

Unusually for a CMIP6 (Coupled Model Intercomparison Project Phase 6) model, UKESM1 simulates full tropospheric-stratospheric chemistry, including ozone, set by the UK Chemistry and Aerosols model, described by Archibald *et al.* (*48*). The ozone field is fully interactive, with its evolution dependent on the model atmosphere’s thermal, dynamical, and chemical states. The ability of UKESM1 to simulate the historical and future evolution of ozone has been discussed in (*49*). Of the 22 models they analyzed, only six interactively predicted stratospheric ozone, UKESM1 being one of these. With respect to global mean total column ozone (TCO), UKESM1 has a positive bias compared to observations for 1980 to 2015. For the antarctic region (60°S to 90°S), this overestimate is significantly reduced. With respect to observed TCO trends, at the global mean, UKESM1 has a stronger negative trend than seen in observations. As for the climatology, this overestimate is significantly reduced when the 60°S to 90°S region is considered. These biases should be kept in mind when the impact of ozone depletion and recovery on the surface physical climate is discussed.

### Scenario description

We consider three scenarios of stratospheric ozone and two scenarios of GHG emissions for a total of six combined stratospheric ozone/GHG atmospheric forcing combinations (fig. S7). Given the key role that ODSs (e.g., chlorofluorocarbons and hydrochlorofluorocarbons) play in driving stratospheric ozone loss (*50*, *51*), we control the evolution of stratospheric ozone in UKESM1 by modifying emissions of ODS. Specifically, we perform simulations for 1950 to 2100 using three ODS emission scenarios: (i) ODSs use standard CMIP6 emissions (historical followed by an SSP). This results in ozone loss (~1970 to 2000) followed by a slow recovery through to 2100, which is typical of the evolution of TCO in CMIP6 models [see figure 7 of (*49*)]. (ii) ODSs are fixed at 1950 values. This minimizes stratospheric ozone loss throughout the simulation as essentially no ODSs reach the model stratosphere. (iii) ODSs are fixed at 1990 values. This leads to maximum ozone loss from 1990 that is maintained through the rest of the simulation.

These experiments are unique in that the ozone scenarios are internally generated by UKESM1 solely on the basis of emissions of ODSs. This means that changes in atmospheric ODS affect both stratospheric ozone and climate more generally, through their direct radiative effect, in an internally consistent manner. This differs from, for example, CMIP6 Detection and Attribution Model Intercomparison Project (*52*), where simulations are run either with, or without, externally prescribed stratospheric ozone concentrations, with no modification of the chemicals (ODS) that drive the ozone loss. While such ozone removal experiments are informative, they are less realistic than the experiments discussed here because (i) no consistent modification is made to the ODS species responsible for the ozone loss, resulting in an incorrect total forcing of the system (e.g., stratospheric ozone is “magically” removed or increased with no change in the chemistry or radiation associated with the drivers of this change), and (ii) artificial removal or addition of stratospheric ozone will result in the host model’s thermal, dynamical, radiative and chemical states no longer being internally consistent. We believe that our approach provides a more realistic and internally consistent protocol for understanding the role of variable stratospheric ozone in the coupled Earth system. Figure S7 shows the evolution of TCO, averaged between 60°S and 90°S in the three ODS scenarios used.

The three ODS scenarios are combined with two CMIP6 SSP scenarios (*35*) that represent a high– and low–GHG emission scenario. We consider two separate SSP scenarios developed to inform the Sixth Assessment Report of the IPCC: the “sustainable” low-emission SSP 1-2.6 scenario and the “regional rivalry” high-emission SSP 3-7.0 scenario (*53*). In the SSP 1-2.6 scenario, decarbonization policies have been largely successful in the context of a world focused on sustainability, and, consequently, atmospheric CO_2_ concentrations peak in the early 2060s at ~475 μatm and, subsequently, decline. In the SSP 3-7.0 scenario, resurgent nationalism and regional conflict inhibit climate change mitigation and adaptation, and the increase of GHG accelerates in the second half of the 20th century, with atmospheric CO_2_ reaching concentrations of ~860 μatm by 2100 (*54*). The two SSP scenarios are chosen to cover the range of plausible futures, given that peak global emissions have not yet been reached, but countries’ actions and latest pledges within the Paris Agreement add up to significant mitigations below the highest SSPs available (*55*).

Combining the three ozone scenarios with the two SSP scenarios allows us to investigate independently the role of changing ozone and changing GHG concentration on the evolution of the system. In all graphs and analyses, we use hue (green, yellow, and red) to denote ozone scenario (no ozone loss, ozone loss and recovery, and ozone loss with no recovery) and brightness (light and dark) to denote carbon scenario [low carbon (SSP 1-2.6) and high carbon (SSP 3-7.0)]. For all scenarios of wind and ocean evolution, we consider the time period 1950–2100.

### Evaluation of UKESM1 wind fields against ERA5

To establish confidence in the UKESM1 representation of wind fields, we evaluate the model’s historical performance for the near-surface wind speed for the time period (1940–2020) against the ERA5 (European Centre for Medium-Range Weather Forecasts Reanalysis v5) reanalysis product from the European Center for Medium-Range Weather Forecasts (*56*). We compare seasonally subdivided climatologies (1940–2020) for the two datasets, trends in seasonally subdivided wind speed magnitude and in the position of the maximum wind speed (the wind jet) in the two datasets, reporting statistically significant linear least-squares regression trends.

Because the UKESM1 and ERA5 10-m wind fields are available at different spatiotemporal resolutions and native grids, we standardize them as follows: We first calculate the 10-m wind speed from its *u* and *v* components at the highest available spatiotemporal resolution for both products, which is 3-hourly for UKESM1 and hourly for ERA5. In the case of UKESM1, where the *u* and *v* components are on slightly different grids, we first interpolate them to a standard 1° × 1° grid using the cdo package. We then calculate a daily average wind speed for both products and then interpolate ERA5 daily winds to the same standard 1° × 1° grid, allowing for direct comparison of the two products. When comparing biases between the two products and mean trends over the historical time period 1940–2020, we consider a seasonal mean, calculated from daily area-weighted mean over-water wind speed values, south of 50°S. Note that seasons are based on a 360° calendar in the UKESM1 model and on a historical calendar in the ERA5 product.

### Comparison of UKESM1 wind fields and trends against ERA5

The UKESM1 model output reasonably reproduces the general spatial structure of the seasonally subdivided climatological (1940–2020) wind speed found in ERA5 (fig. S8), characterized by a prominent, well-known wide band of fast winds between 40° and 60° south that reach a maximum in the Indian Sector and lower wind speeds at high latitudes near the continent. The general seasonality is also consistent between the products, with winds reaching a climatological maximum in austral winter (June-July-August) and a climatological minimum in austral summer (DJF) in most regions in both products. The UKESM1 product tends to slightly underestimate the ERA5 winds at high latitudes near the continental shelf (south of 50°S) and overestimate them substantially at subantarctic latitudes over the open ocean (50°S to 30°S) (table S2). The overall bias of the UKESM1 climatology against the ERA5 product (calculated from the 1° × 1° regridded products) is then small, and we find good agreement between the two products in the entire area south of 50°S, with a full-year bias of −0.036 m s^−1^ (UKESM-ERA) that reaches a maximum in austral summer (DJF) at −0.100 m s^−1^. Because of this good agreement, here, we focus our analysis of wind speed evolution and its effects on ocean state on the area south of 50°S.

Both products report no significant increases in wind speed for the first half of the climatology (1940–1980), including a slight decrease in March-April-May (MAM) in ERA5 and significant increases in the time period 1980–2020 that are somewhat stronger in the UKESM1 product (fig. S9 and table S3). Over the whole time series 1940–2020, trends are significant in all seasons but strongest in austral summer (DJF; fig. S4 and table S3), and the agreement in wind speed trend tendency between the two datasets is good, especially in austral summer and austral autumn (MAM). In the other two seasons, the ERA5 trends are somewhat stronger for the entire time period 1940–2020.

We expect to see a poleward shift in the polar jet in conjunction with the depletion of stratospheric ozone in the last decades of the 20th century (*23*). Here, we formalize the jet position as the location of the maximum zonally averaged wind speed south of 30°S, which we calculate at daily resolution. From daily resolution jet position, we calculate seasonal means for each year and then calculate seasonally subdivided trends (fig. S9 and table S4). The two products agree best in absolute position and trend in austral summer (DJF; table S4). In other seasons, the UKESM1 jet is somewhat further north (1° to 2° latitude) than the ERA5 one and also shows a more pronounced poleward migration. In both products, no statistically significant migration of the jet is seen in any season in the time period 1940–1980, and an overall poleward migration is seen in austral summer over the whole 1940–2020 time series; this summer migration of the jet is nearly twice as strong in UKESM1 as in ERA5. The poleward movement is typically stronger in UKESM1 overall and, unlike in the ERA5 reanalysis, is seen to be statistically significant in the latter half of the time series in both summer and winter.

### Estimation of effect of ozone and GHG trends on changes in wind and ocean fields

We show time series of seasonally and yearly averaged SST, MLD, MOC, and air-sea CO_2_ flux, south of 50°S (Fig. 2 and fig. S3). MLD is defined by a Δσ_θ_ criterion of 0.01 with respect to σ_θ_ at 10 m depth. For the MOC, we calculate the maximum of yearly and seasonally averaged σ-coordinates overturning at or south of 50°S in each UKESM1 simulation.

To obtain the relative contribution of ozone to winds, SST, MLD, and MOC, we make the assumption that the response of each field is additive (*24*) (that is, the total change in a field is equal to the sum of the change due to ozone changes and the change due to GHG changes) and subtract the time series from the “no–ozone loss” scenario from the “ozone-loss and recovery” scenario under both SSP scenarios. We use the no–ozone loss scenarios to estimate changes to all fields due only to GHG loading, treating the mean of years 1950–1960 as a baseline and assuming that the effects of GHG loading on the fields of interest are small before 1950 {e.g., [(SSP 1-2.6, no ozone loss) – (SSP 1-2.6, no ozone loss_(1950–1960)_)] gives the evolution of a field only because of GHG forcing under the SSP1-2.6 scenario}. These estimates may be complicated by differing internal and decadal variability between individual scenarios, making small effect sizes hard to discern but, nevertheless yield a useful comparison of the relative magnitudes and seasonalities of the different effects. Comparing the effect of ozone isolated from SSP 1-2.6 and SSP 3-7.0 scenarios confirms that the two effects are additive, for example, wind speed changes due to changing ozone levels isolated from high- and low-carbon scenarios are of similar magnitude (Fig. 1, C and D).

We then calculate linear trends in the time series thus obtained for the time periods 1950–2000, 2000–2050, and 2050–2100 to get trends in the wind and ocean fields. From these trends, we calculate the 50-year contribution of both ozone changes and GHG loading to the changes in these quantities (δSST, δMLD, and δMOC; Fig. 3 and table S5). [For the continuous time series of climate feedbacks shown in Fig. 5, δSST, δMLD, and δMOC are year-on-year total changes in SST, MLD, and MOC, under the most realistic ozone scenario (ozone loss and recovery), not subdivided into their ozone- and GHG-driven components.]

### Estimation of dynamical contributions to changes in the surface DIC concentration

We estimate the individual contributions of MLD, SST, and MOC due to ozone and GHGs calculated above (δSST, δMLD, and δMOC; table S5) to changes in the surface DIC concentration (or, in the case of SST, to a reduction in surface DIC capacity at equilibrium) based on known relationships relating solubility and ocean dynamics to DIC described below. To test the robustness of our results and avoid known biases, we perform the same calculation twice with two different data sources: The first calculation uses observationally constrained biogeochemical datasets, while the second uses the fields and UKESM1 biogeochemical model, MEDUSA. We lastly perform a sensitivity test using artificially strong DIC depth gradients.

Here, we first describe the method, which is not reliant on the data source used. We then describe the data sources. We lastly outline key biases known in the UKESM1 MEDUSA model, as well as the choices and assumptions made when using observationally constrained values, and their potential effects on the results.

### Contribution of MLD to changes in surface DIC

We consider two components of the influence of changes in the MLD on the surface DIC: changes in DIC caused by changes in the entrainment of DIC-rich deep waters to the surface and changes caused by changes in iron delivery to the surface and its subsequent effect on the biological surface carbon uptake and drawdown.

The change in DIC (in micromolar) due to entrainment is

$$\text{δDIC}=\text{δMLD}\cdot \frac{\text{δDIC}}{\delta z}$$ (1)

where $\frac{\text{δDIC}}{\delta z}$ is the DIC gradient (in micromolar per meter) over the top 200 m. A depth horizon of 200 m is chosen here as it is generally below the winter MLD.

To calculate the change in DIC due to changes in primary productivity induced by iron fertilization, we first calculate the change in surface iron concentration due to MLD changes

$$\delta \mathrm{Fe}=\text{δMLD}\cdot \frac{\delta \mathrm{Fe}}{\delta z}$$ (2)

We then estimate the resulting change in DIC by calculating enhanced primary production stimulated by Fe, following a growth formulation that involves nutrients and light limitation (*57*, *58*)

$$\text{δDIC}=-1\cdot \frac{\delta \mu }{\delta \mathrm{Fe}}\cdot \delta \mathrm{Fe}\cdot \mu L\cdot \text{DIAT}\cdot 365.25\cdot f$$ (3)

where $\frac{\delta \mu }{\delta \mathrm{Fe}}$ is the change in growth rate due to changes in iron (0.59 day^−1^ nmol of Fe liter^−1^), estimated from a linear trend for values of Fe < 0.4 nmol of Fe liter^−1^, using a *K*-half of 0.35 nM (*59*) and a mean growth rate for low temperatures of 0.44 1/day (*58*); μ*L* is the dimensionless light limitation coefficient, for which we use a yearly average value of 0.44, derived from the seasonality of open-water daily mean surface solar radiation south of 50°S (*56*) (where a value of 1 represents maximum solar radiation); DIAT is the yearly mean diatom concentration, which is used here as its high growth rate means that it will likely outcompete other types of plankton in the Southern Ocean; 365.35 is a conversion from day^−1^ to year^−1^; and the *f* ratio is the fraction of the primary production that is assumed to sink to the intermediate and deep ocean. We use a relatively high f ratio of 0.5, consistent with high-latitude cold waters (*60*).

### Contribution of overturning to changes in surface DIC

We make the assumption that waters in the surface clockwise overturning cell at or below 50°S are ventilated south of 50°S, bringing extra DIC from depth to surface

$$\text{δDIC}=\text{δMOC}\cdot \Delta {\text{DIC}}_{\text{deep-surface}}$$ (4)

This extra DIC (δDIC) is then assumed to be distributed over the area of the Southern Ocean and depth of the mixed layer to obtain a surface-layer concentration change. For the deep DIC, we take the nominal value at 1000 m, but variations in DIC with depth are small in this depth range (~500 to 2000 m). We proceed similarly as in Eq. 4 to estimate a surface Fe change

$$\mathrm{\delta Fe}=\text{δMOC}\cdot \Delta {\text{Fe}}_{\text{deep-surface}}$$ (5)

which we then convert to a surface DIC change as in the case with MLD (Eq. 3).

### Contribution of SST change to changes in surface DIC capacity

The warming of the surface ocean decreases the solubility of DIC, with pCO_2_ increasing ~4% per degree of warming (*13*), and, as the uptake of DIC is given by the air-sea disequilibrium, as pCO_2_ increases with temperature, the surface ocean can hold less DIC for a given pCO_2_. We translate a pCO_2_ change with temperature into a DIC change by subtracting the DIC corresponding to the initial pCO_2_ from the DIC corresponding to the pCO_2_ altered by temperature changes, assuming that other carbonate chemistry parameters [alkalinity and *S* (salinity)] remain constant. We use the mocsy package for all carbonate chemistry calculations (*61*).

### Observational and model biogeochemical values used, biases, and assumptions

When performing the above calculation with the UKESM1 MEDUSA model, all necessary values are available as model output; for the observationally constrained calculation, we use a range of datasets, which we detail in table S6, where we also summarize the biogeochemical values used in both the observationally constrained and model-derived calculation. We also show both the UKESM1 MEDUSA model–derived biogeochemical values and the observationally constrained biogeochemical values in fig. S4. Here, we provide more detail on the observational datasets used. We then discuss known biases in the model, as well as the choices and assumptions made when using the observationally constrained values. For clarity, we summarize these biases and assumptions in table S7.

### Observationally constrained datasets—Iron and diatoms

To obtain observational iron values for use in the MLD and MOC calculations, we use the median of observations from the GEOTRACES 2021 Intermediate Data Product (IDP2021) (fig. S10) (*62*). We use the latest (2016) update of the mean diatom concentration observations from MAREDAT (*63*), weighted by season (the annual mean is the mean of the seasonal means) (fig. S11).

### Observationally constrained datasets—Carbonate chemistry

We obtain a mean DIC, anthropogenic DIC, and total alkalinity (TA) depth profile from the GLODAPv2 gridded product (*64*), which are standardized for year 2002. We then calculate an observationally constrained time-varying DIC profile using surface DIC and TA from GLODAPv2 and calculating oceanic surface pCO_2_ for year 2002 with *T* (temperature) and *S* from the model for the corresponding year. The *T* and *S* from the physical model are used here so the effects of changing *T* and *S* on carbonate variables can be taken into account on the basis of the UKESM1 projections. Using the atmospheric pCO_2_ for 2002, we can get a ΔpCO_2_ that we assume is approximately constant for the full time series. Then, for each year during 1950–2100, we use the atmospheric pCO_2_ and the constant ΔpCO_2_ derived initially to get an oceanic pCO_2_, from which we can calculate a surface DIC using time-varying model *T* and *S* and the 2002 GLODAPv2 TA. We assume that the proportion of anthropogenic DIC at depth to DIC at surface remains constant, so we can use the depth profile of anthropogenic DIC at year 2002 and the reconstructed surface DIC time series for 1950–2100 to obtain time-varying DIC profiles for the entire time series (fig. S4).

### Known biases in the MEDUSA model

Like many biogeochemical models, MEDUSA is generally diffusive (*40*), and, as a result, it tends to underestimate vertical gradients in biogeochemical tracers. For example, the mean vertical surface DIC gradient of MEDUSA over the historical period 1950–2000 is substantially lower than that estimated from the GLODAPv2 observations (0.22 μM m^−1^ in the top 200 m, as opposed to 0.41 μM m^−1^ in GLODAPv2; fig. S4). This lower gradient means that any changes in the surface DIC balance from changes in the MLD or overturning will be reduced, as deeper water in the model is comparatively more similar to surface water. MEDUSA also has a lower surface diatom concentration than the observations, likely leading to a reduced effect of changes in biological activity on the surface DIC balance (table S6 and fig. S4). Last, MEDUSA has a substantially lower difference between surface and deep iron concentrations than is seen in the observations, which also leads to a reduced effect of changes in biological activity (due to iron delivery from increased overturning) on the surface DIC balance. However, the contribution of biological activity to changes in the surface DIC balance is small to begin with in the observations. These biases are summarized in table S7.

### Assumptions when using observationally constrained values

When obtaining future surface DIC values and DIC gradients from the existing historical GLODAP observations, we are effectively isolating the effect of rising atmospheric CO_2_ concentration on the surface DIC balance and ignoring the effects of changes in circulation, biological activity, and TA, which are included in the MEDUSA model. Here, we list the assumptions that we are making. First, we assume that TA is constant. In reality, TA could decrease in the future, for example, because of melting sea ice, which would decrease the efficiency of the ocean in taking up DIC, meaning that the future estimates of surface DIC derived from GLODAP would likely be too large, which we see in fig. S4. (In the model, TA decreases by ~16 μM from 2015–2025 to 2090–2100 under SSP 3-7.0.) We also assume that the diatom biomass and iron distributions, whose contributions to changes in the surface DIC balance are small in the historical time period, stay constant throughout the time series.

Next, we assume that the ΔpCO_2_ calculated from the historical observations remains relatively constant (that is, oceanic pCO_2_ linearly follows atmospheric pCO_2_). In reality, oceanic surface pCO_2_ is affected by all of the processes discussed here and changes in response to changes in temperature, TA, circulation, or biological activity. However, changes in ΔpCO_2_ in the UKESM1 model, which represents all of the above processes, are small relative to changes induced by the rise in atmospheric CO_2_, with a decrease of ~17 μM from 2015–2025 to 2090–2100 under SSP3-7.0, which translates to a ~6 μM decrease in DIC when compared to the case with constant ΔpCO_2_ or ~8% of the total change in DIC (driven primarily by the rise in atmospheric CO_2_) over that time period. Last, we assume that the shape of the anthropogenic DIC gradient stays constant, which is to say, if the anthropogenic DIC load at depth *D* was *P*% of the anthropogenic DIC load at the surface in year 2002, then this percentage *P* is constant in time, which could again change in either direction due to changes in circulation.

Because of these assumptions, summarized in table S7, these surface DIC extrapolations are not expected to follow the model exactly. Specifically, the assumption that both TA and ΔpCO_2_ are constant will tend to lead to an overestimation of the DIC increase, which is visible in fig. S4. However, the surface DIC time series obtained this way is relatively similar to the one obtained from MEDUSA (fig. S4) because the increase in anthropogenic atmospheric CO_2_ remains the most important driver of the DIC increase.

### Sensitivity test and effect of known biases and assumptions

In addition to the two calculations described above, we do a sensitivity test of the carbon-climate feedbacks with artificially high DIC gradients. Specifically, we double the observational $\frac{\text{δDIC}}{\delta z}$ surface gradient over the top 200 m and add 100 μM DIC to the observational ΔDIC_deep-surface_ to test the system under artificially strong DIC depth gradients (fig. S6). We do not change the observational iron and biological values, which contribute little to the carbon-climate feedbacks (Figs. 4 and 5).

Estimates of the cumulative climate feedbacks under the SSP3-7.0 scenario show similar behavior when using all three sets of biogeochemical parameters (Fig. 5 and fig. S6): Only the SST feedback grows over the course of the 21st century, while the cumulative contributions of changes in overturning and MLD, which typically oppose each other, stagnate as DIC gradients with depth diminish, leading to clear overall reduction in the carbon-climate feedback over the 21st century in all three cases. Because the method used to extrapolate DIC to the future tends to overestimate future DIC, the proportional carbon-climate feedbacks are smaller in the observationally constrained estimate than in the model one (Fig. 5 and fig. S6). However, the general trend of diminishing carbon-climate feedbacks over the 21st century, which becomes dominated by the effect of warming, clearly holds under all three calculations (Fig. 5 and fig. S6), suggesting that our conclusions here are robust to the choice of method and to the uncertainty in biogeochemical values.

### DIC-SST feedback estimation

We also calculate a cumulative DIC-SST feedback, calculated as ΔDIC_feedback_/ΔSST time series, where ΔSST_year *X*_ = SST_year *x*_ − SST_1950_ and analogously for DIC (Fig. 5A and fig. S6) for the future projection under SSP3-7.0 (2015–2100). (The initial increase in ΔDIC_feedback_/ΔSST at the beginning of the future projection is due to a temporary SST decline in this run from 2015–2025.) This feedback declines over the course of the 21st century as the ocean carbon sink becomes less effective.
