# Supplementary material for: Decreasing importance of carbon-climate feedbacks in the Southern Ocean in a warming climate
Source: Sci Adv. 2025 May 16;11(20):eadr3589. doi: 10.1126/sciadv.adr3589 (PMC12083540; doi:10.1126/sciadv.adr3589)
Supplement: Supplementary file 1 — Figs. S1 to S11 Tables S1 to S7 [file sciadv.adr3589_sm.pdf]

Science Advances
AAAS

Supplementary Materials for

Decreasing importance of carbon-climate feedbacks in the Southern Ocean in a warming climate

Tereza Jarníková *et al.*

Corresponding author: Tereza Jarníková, t.jarnikova@uea.ac.uk

Sci. Adv. **11**, eadr3589 (2025)
DOI: 10.1126/sciadv.adr3589

This PDF file includes:

Figs. S1 to S11
Tables S1 to S7

Supplementary material

Supplementary Figures

Fig. S1

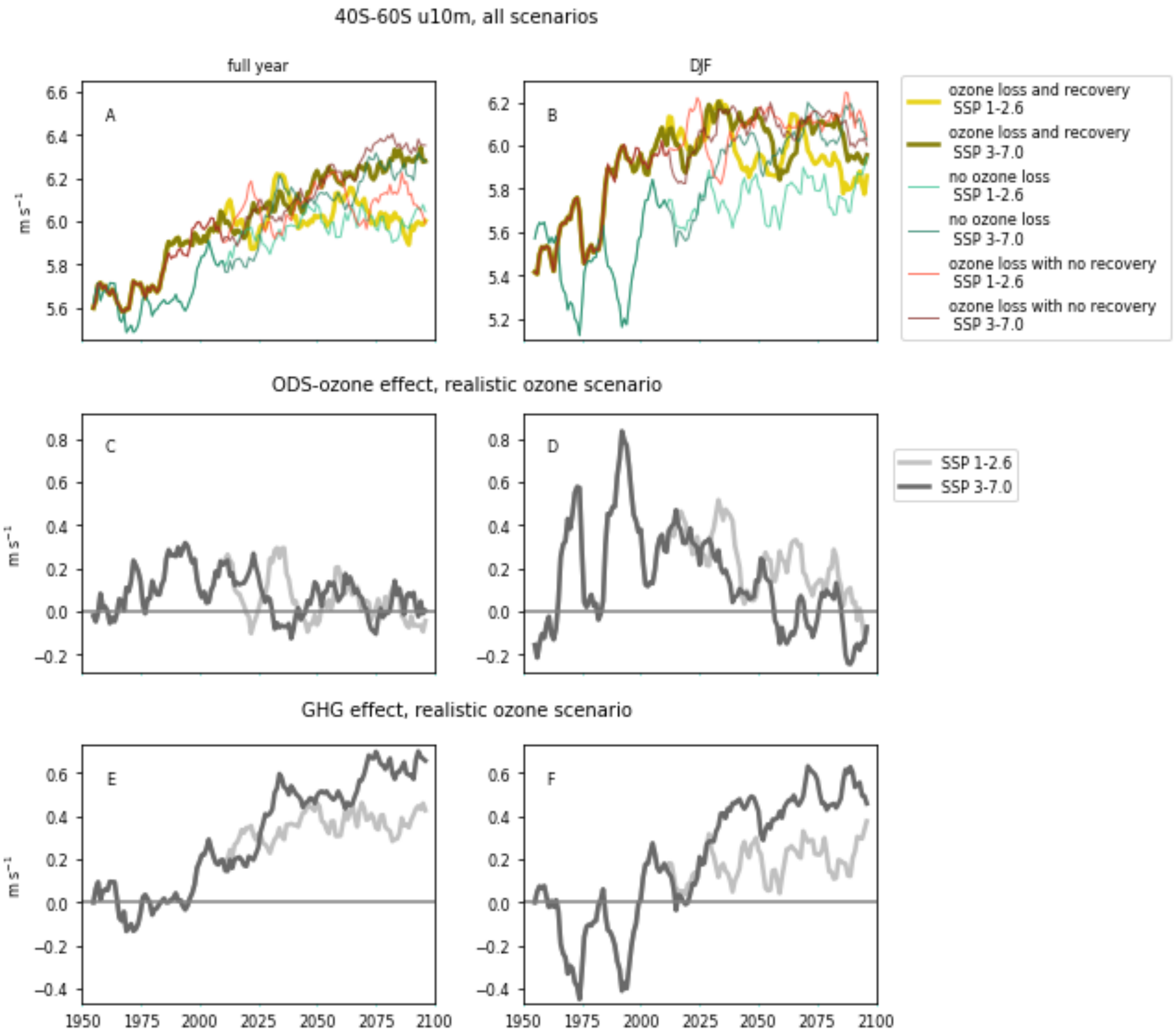

Fig. S1. Southern Ocean mean u-component of open-water wind speed at 10m (u10m), between 40°S-60°S 1950-2100 (m s^{-1}). Year-round (left) and austral summer (DJF) (right) mean u10m for all 6 scenarios conducted with the UKESM1 (a,b), as well as a decomposition of the contribution of ODS changes (c,d) and GHG changes (e,f) to wind speed trends under the realistic ozone scenario (with depletion to 1990 and recovery thereafter) and the two GHG scenarios (SSP1-2.6 and SSP3-7.0). Timeseries are smoothed with a 10-year running mean.

Fig. S2

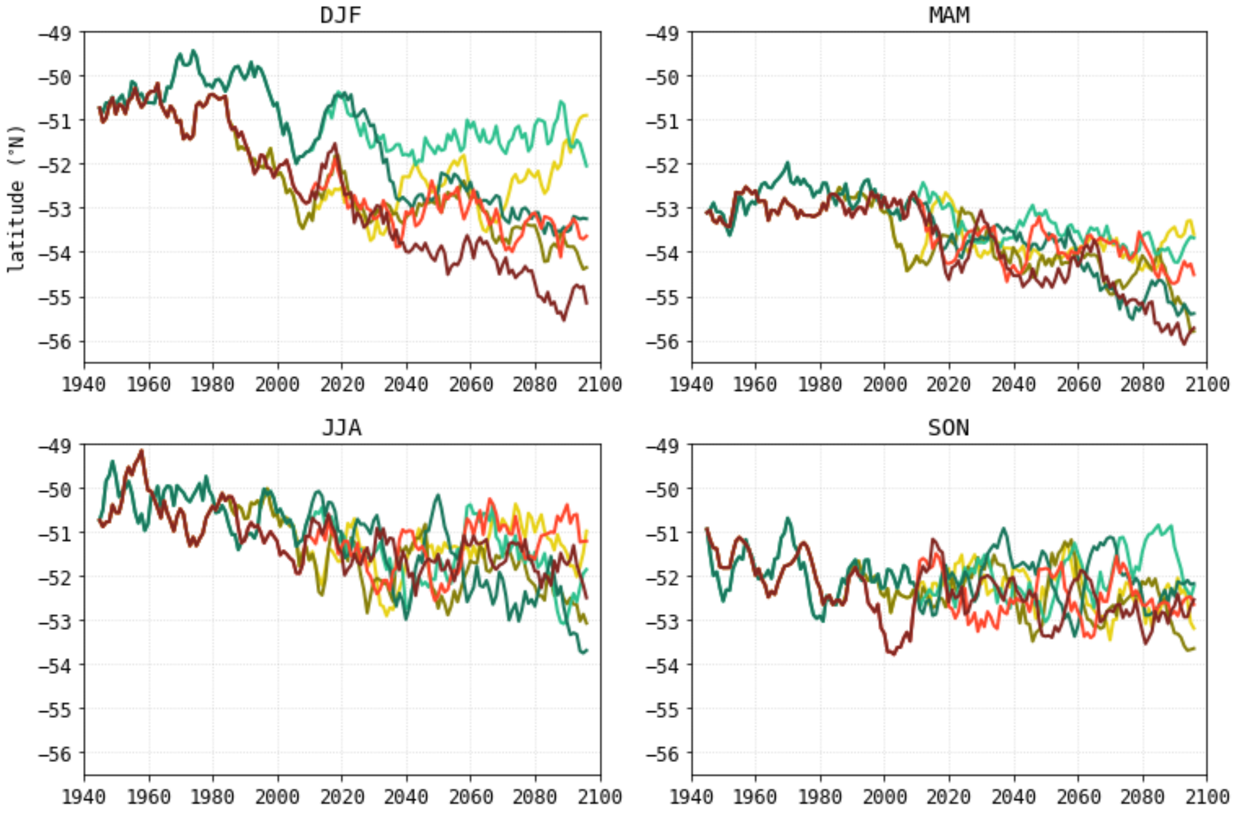

Fig. S2. Position of the wind jet in the 6 scenarios over 1950-2100. (See Fig 1, fig S7 for scenario colour descriptions). Timeseries are smoothed with a 10-year running mean.

Fig. S3

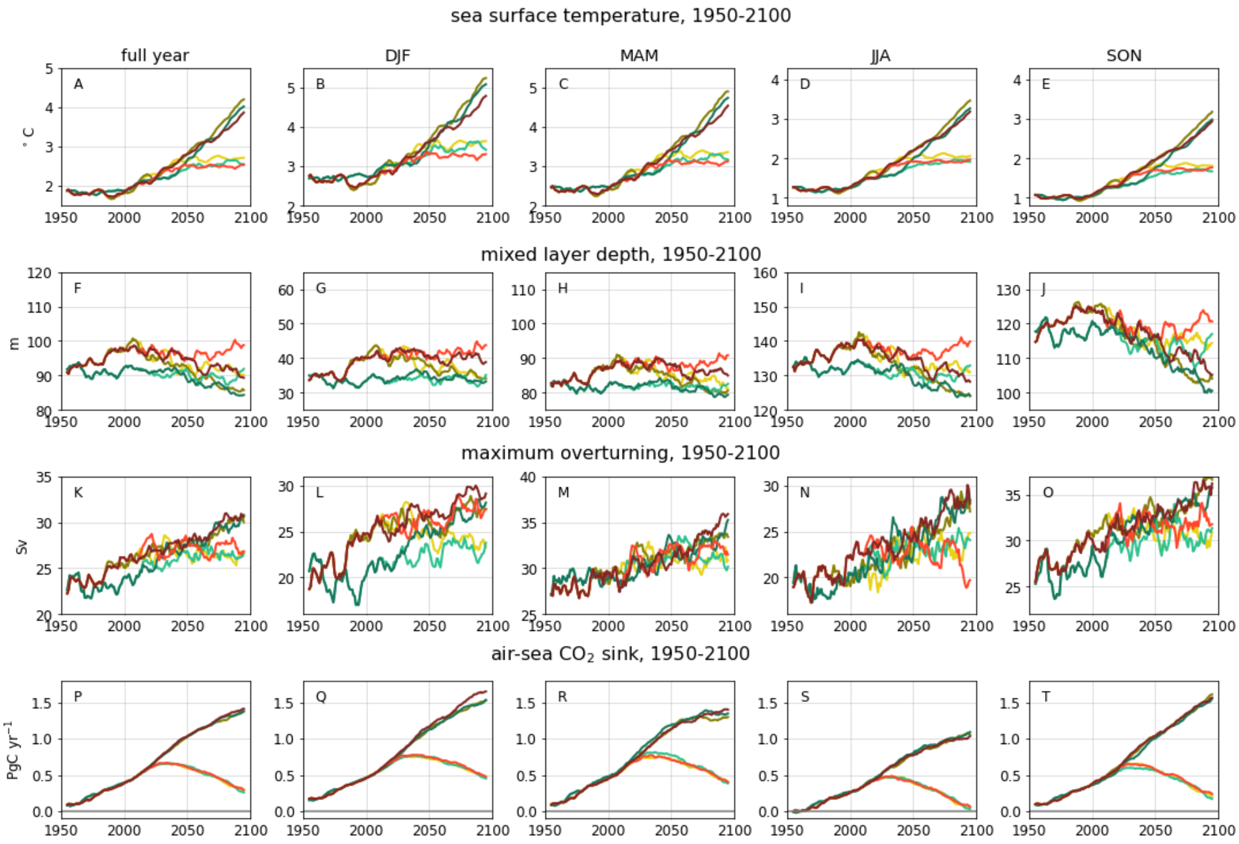

Fig. S3. Yearly-average and seasonally subdivided time series of SST (panels a-e), MLD (panels f-j), maximum σ-coordinates MOC (panels k-o), and air-sea CO_2 sink (panels p-t). See also Fig. 2. For SST, MLD, and air-sea CO_2 sink, values shown are averages south of 50°S. For MOC, the value given is the maximum of the yearly average overturning at or below 50°S. Timeseries are smoothed with a 10-year running mean.

Fig. S4

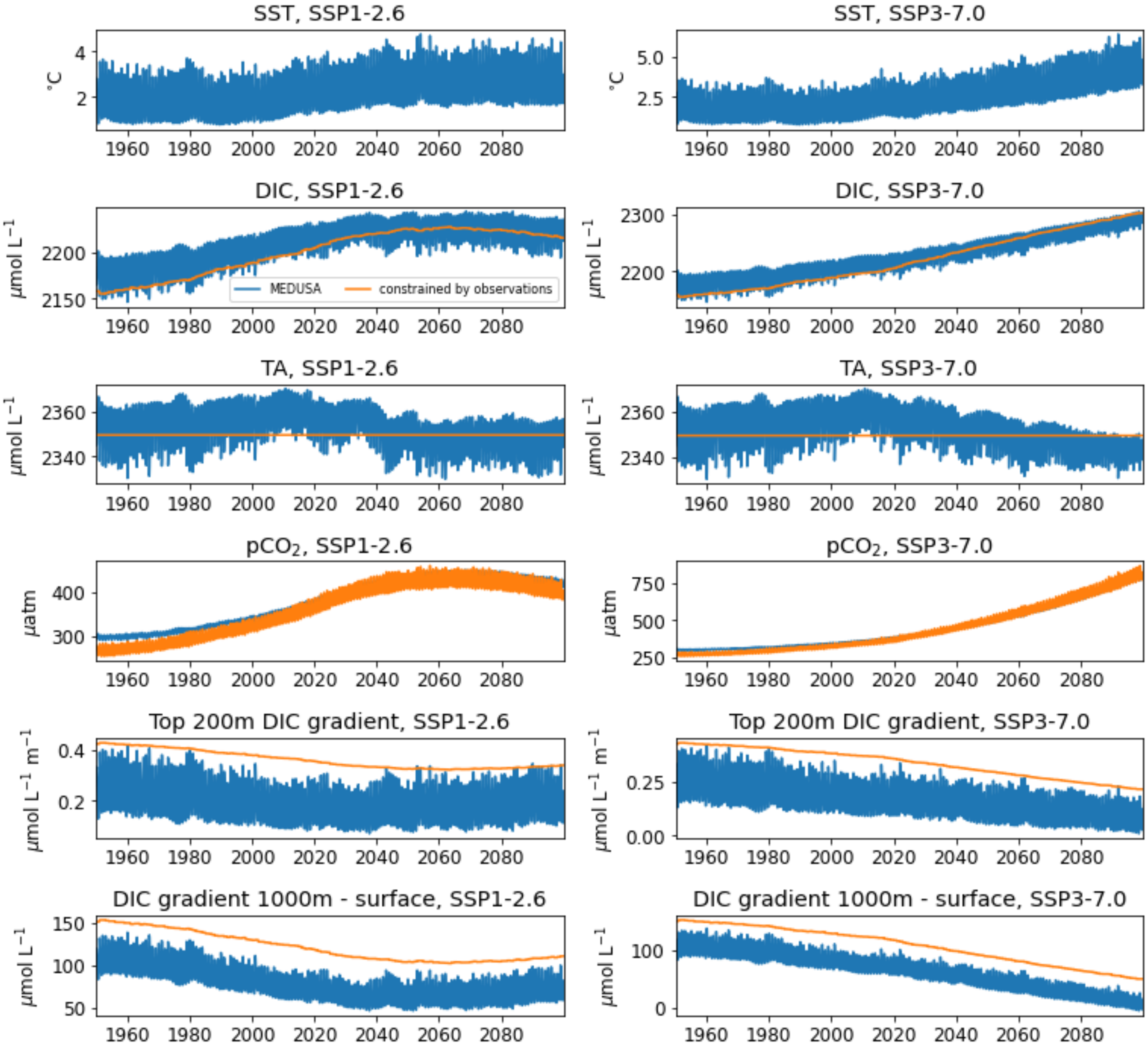

Fig. S4. Timeseries of carbonate chemistry parameters from the UKESM1 MEDUSA model (blue) and those extrapolated from present-day observations (orange). For future values for the parameters based on present-day datasets, see Methods, sections "Observationally-constrained datasets – carbonate chemistry" and "Assumptions when using observationally-constrained values".

Fig. S5

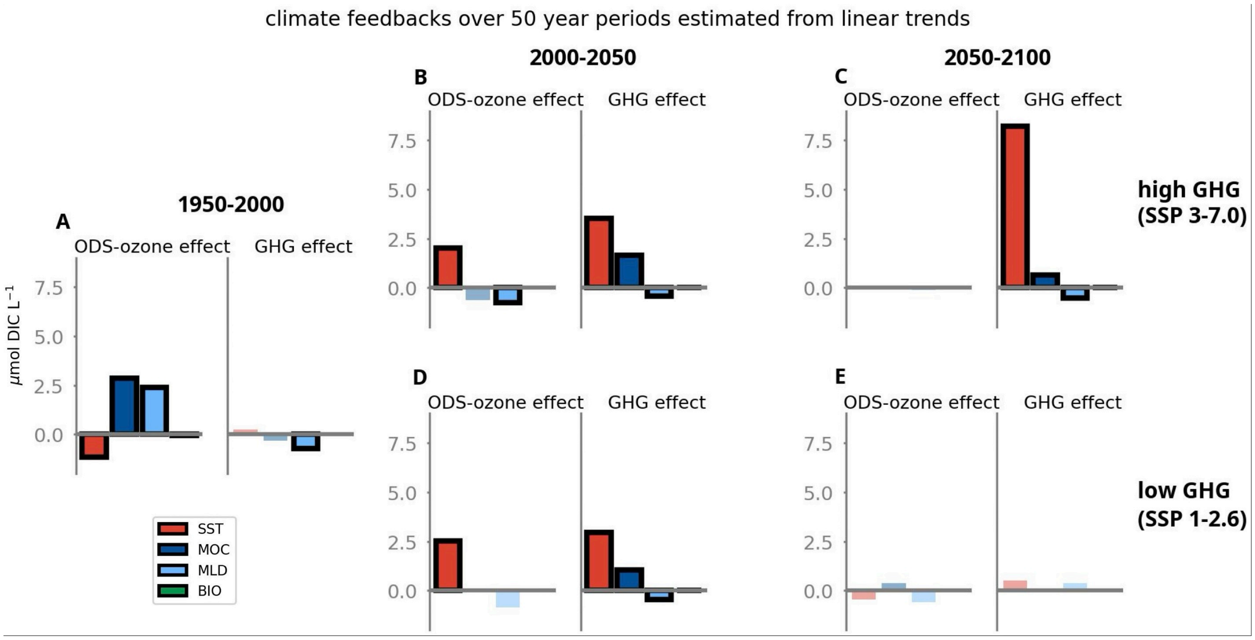

Fig. S5: Effective contribution of ozone-forced (left) and GHG-forced (right) climate feedbacks from SST, MOC, MLD, and biological carbon drawdown (BIO) to mean changes in surface Southern Ocean DIC concentrations (south of 50°S), using biogeochemical values derived from the UKESM1 MEDUSA model (see Methods). Panel a): historical time period 1950-2000, panel b): 2000-2050, SSP3-7.0, panel c): 2050-2100, SSP3-7.0, panel d): 2000-2050, SSP1-2.6, panel e): 2050-2100, SSP1-2.6. For a version of this figure with observationally-derived biogeochemical values, see Fig. 4.

Fig. S6

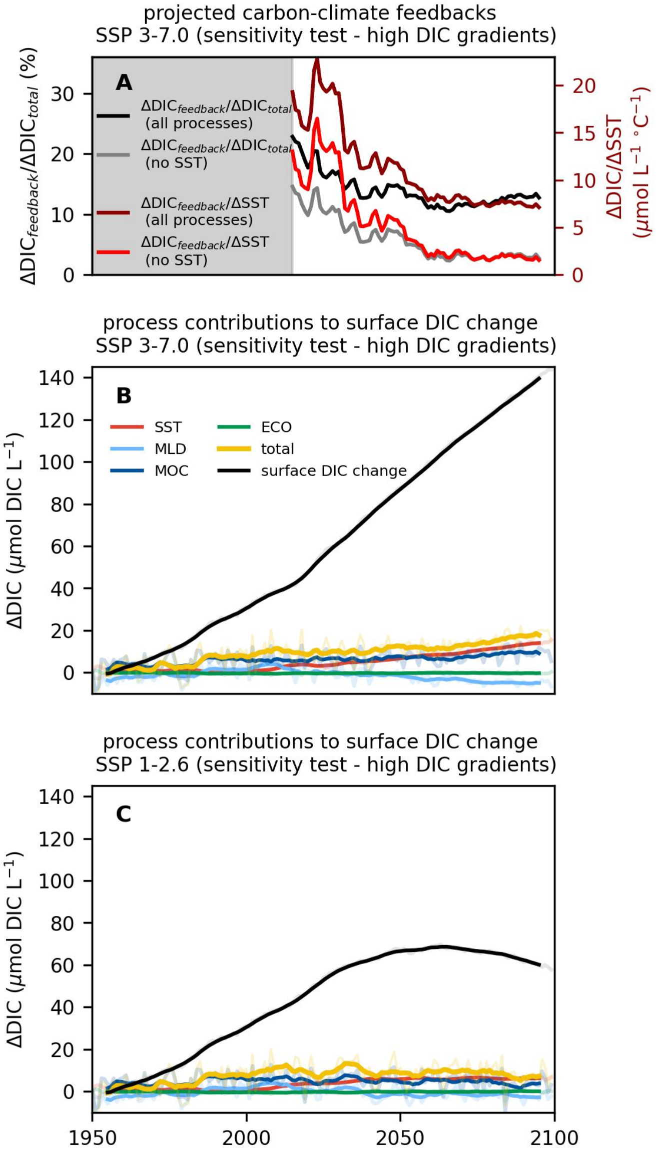

Fig. S6. Sensitivity test of effective contribution of total climate feedbacks from continuous timeseries of SST, MOC, and MLD to changes in surface Southern Ocean DIC concentrations (south of 50°S) under SSP 3-7.0 (b) and SSP 1-2.6 (c), using artificially high DIC gradients with depth (see Methods and Fig. 5 in main text). Panel a): Projected cumulative carbon-climate feedbacks in the Southern Ocean under a high emission scenario (SSP3-7.0). Left axis: black line represents (cumulative process-driven changes in DIC, all processes)/(total change in DIC) while grey line represents (as above excluding SST effects)/(total change in DIC), right axis: the maroon line shows (cumulative process-driven changes in DIC, all processes)/(cumulative change in SST) while the red line shows (as above excluding SST effects)/(cumulative change in SST). Panels b and c): Cumulative changes in surface DIC concentrations broken down into the effective cumulative contribution of changes in

individual processes in the Southern Ocean (μmol L^{-1}) under high (SSP3-7.0) (b) and low (SSP1-2.6) (c) emission scenarios.

Fig. S7

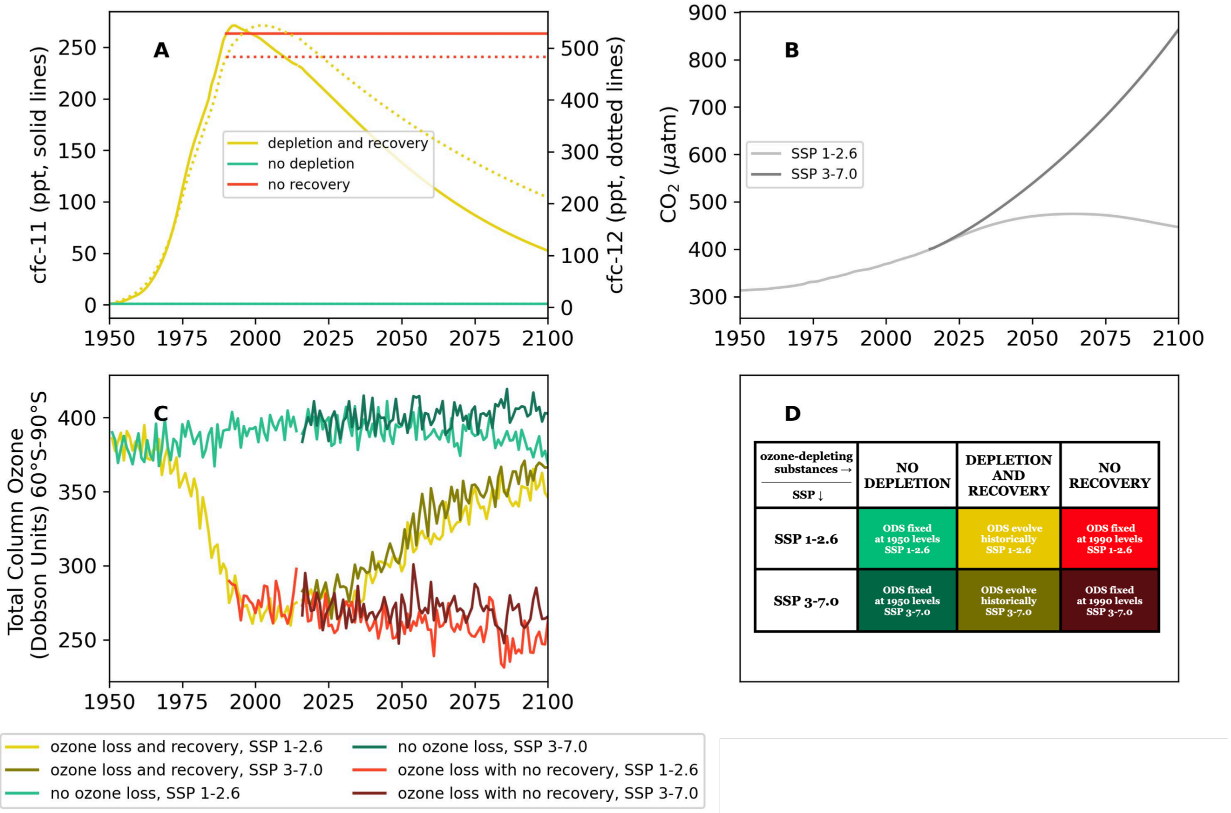

Fig. S7. Levels of ODS (cfc-11 and cfc-12, full and dotted lines respectively, panel a), CO_2 (panel b), and TCO (60°S-90°S, panel c) by scenario. A schematic of the six combinations of scenarios, with associated colours, is given in panel d.

Fig. S8

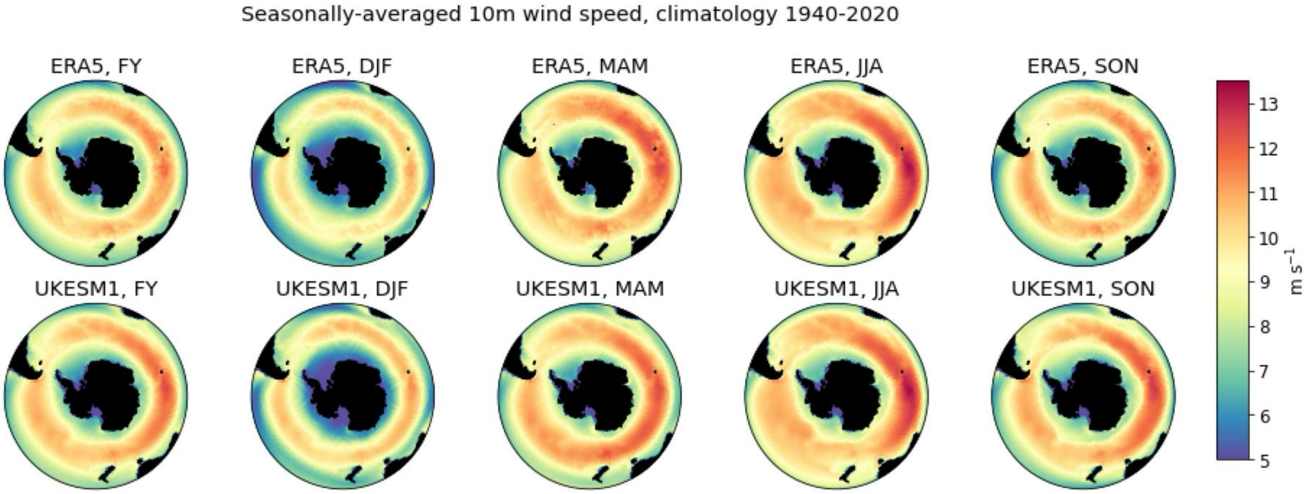

Fig. S8. Seasonally-averaged climatological mean 10-m wind speed fields for the UKESM1 model and the ERA5 reanalysis product (1940-2020).

Fig. S9

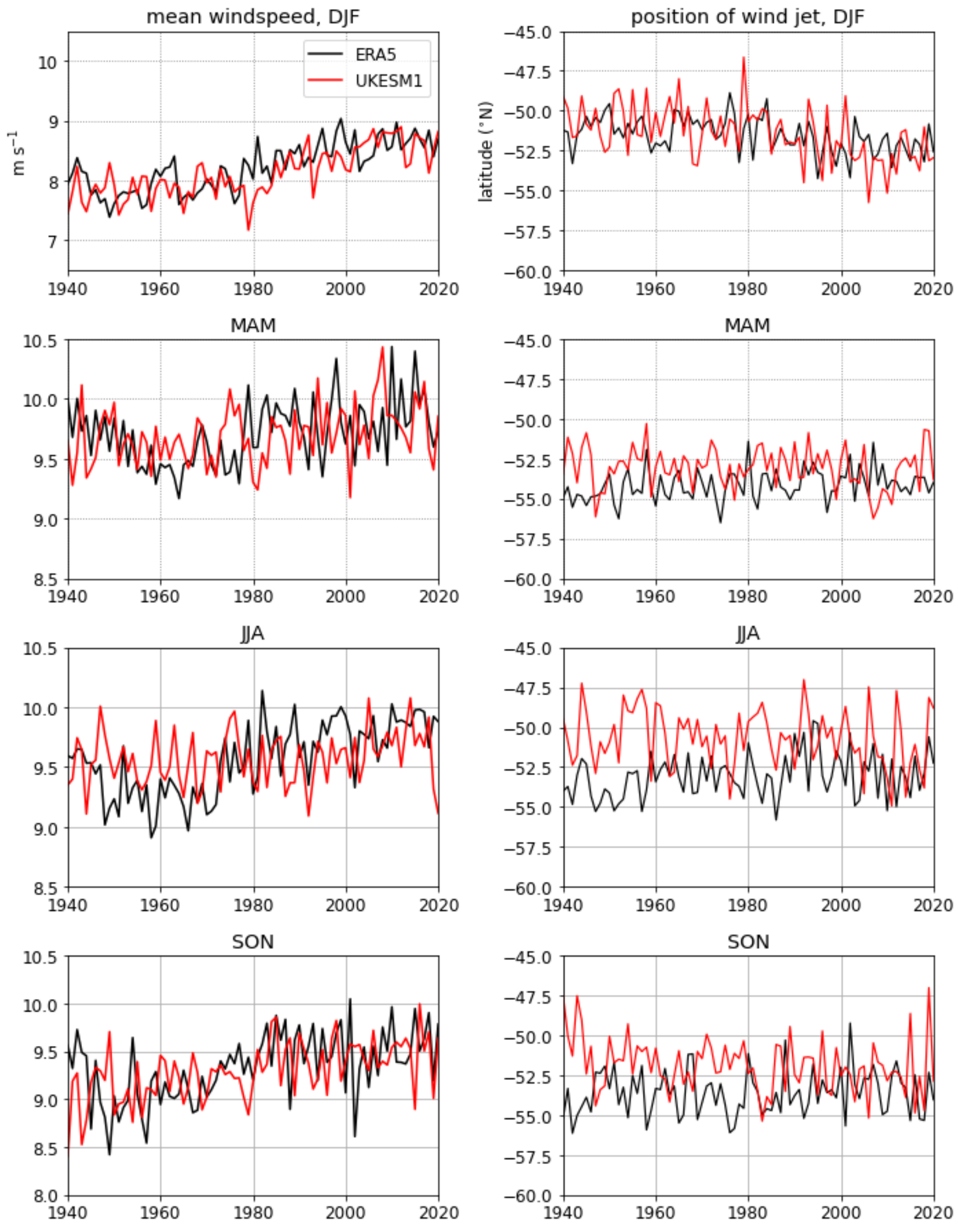

Fig. S9. Seasonally-subdivided mean overwater wind speed (left column) and mean wind jet position (right column) from UKESM1 and the ERA5 reanalysis south of 50°S.

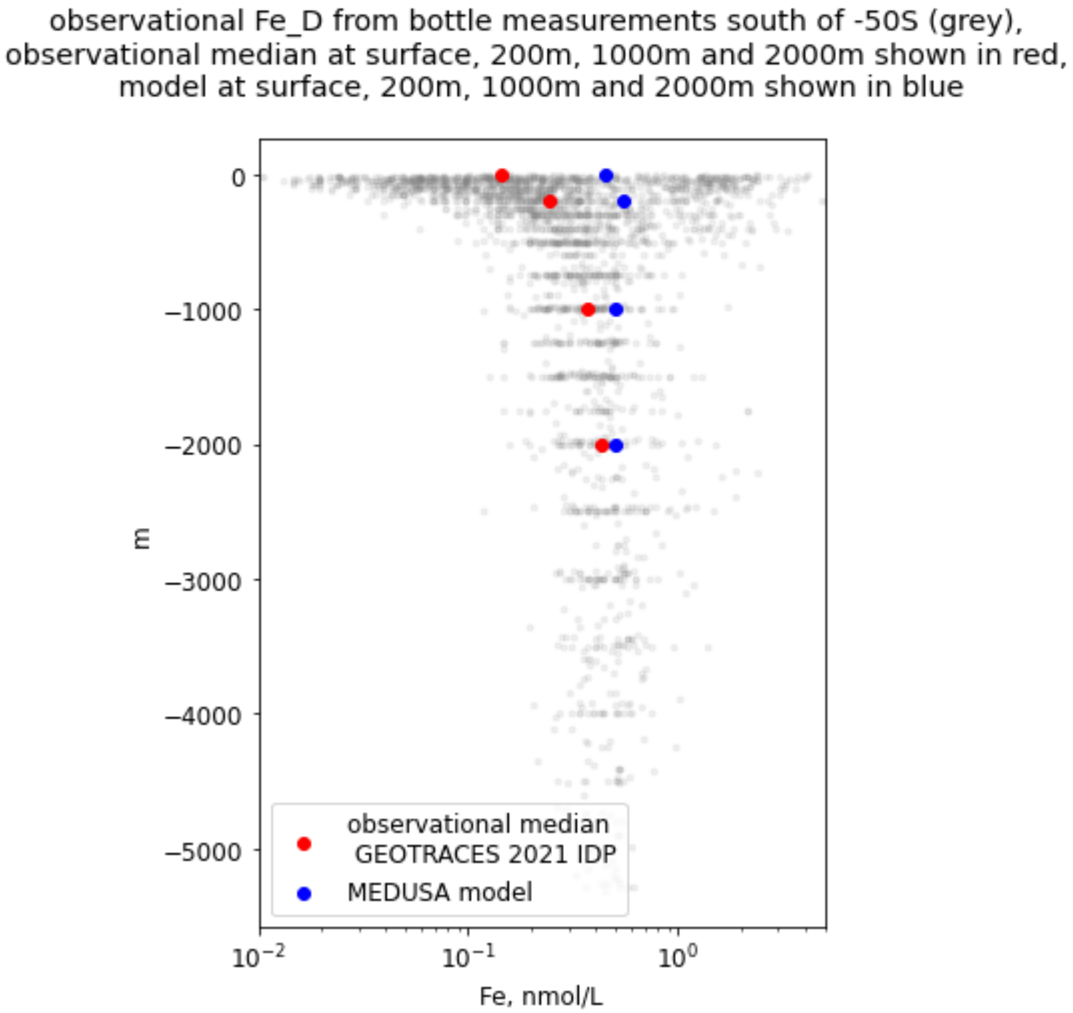

Fig. S10

Fig. S10. Southern Ocean (south of 50°S) dissolved iron concentrations from the GEOTRACES database. Values from the UKESM1 MEDUSA model are overlaid for comparison.

Fig. S11

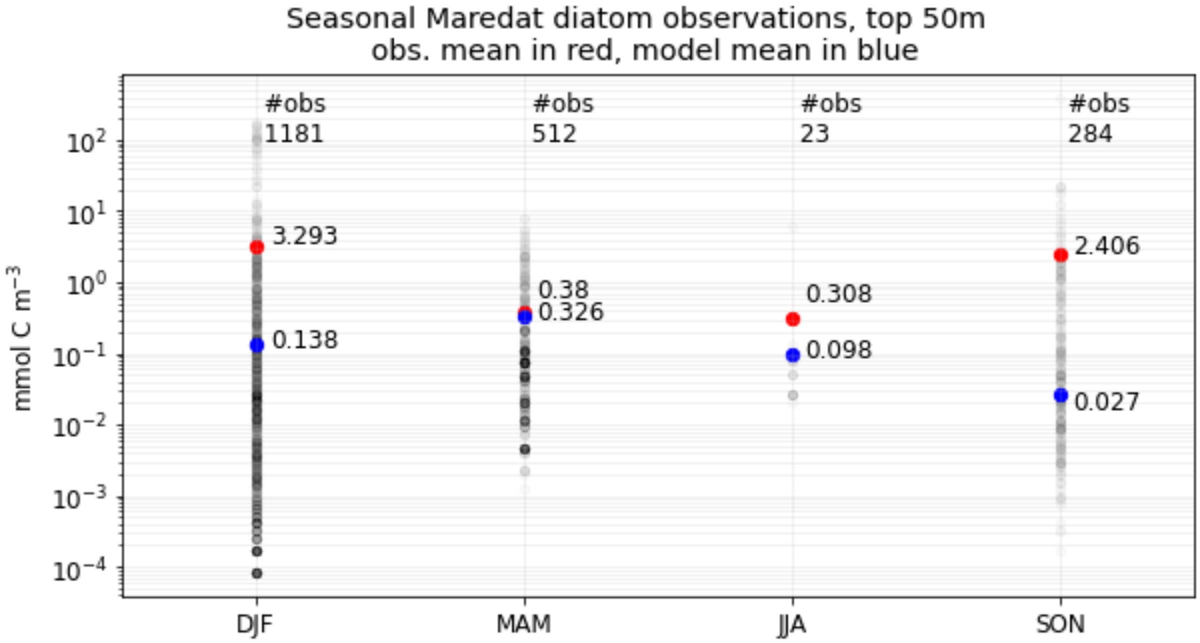

Fig. S11. Seasonally subdivided Southern Ocean (south of 50°S) diatom concentrations from the MAREDAT database. The observational mean is shown in red and the UKESM1 MEDUSA mean is shown in blue.

Supplementary Tables

table S1

	SSP1 no ozone loss	SSP1 ozone loss and recovery	SSP1 ozone loss with no recovery	SSP3 no ozone loss	SSP3 ozone loss and recovery	SSP3 ozone loss with no recovery
	1950-2100 decadal trends in wind speed (m s^{-1})					
**FY**	0.028	0.028	0.044	0.051	0.05	0.062
**DJF**	0.036	0.035	0.083	0.071	0.07	0.105
**MAM**	0.027	0.025	0.036	0.046	0.045	0.057
**JJA**	0.027	0.023	0.018	0.045	0.039	0.039
**SON**	0.023	0.027	0.037	0.04	0.045	0.048
	1950-2000 decadal trends in wind speed (m s^{-1})					
**FY**	--	0.063	0.067	--	0.063	0.067
**DJF**	--	0.122	0.127	--	0.122	0.127
**MAM**	--	--	--	--	--	--
**JJA**	--	--	--	--	--	--
**SON**	--	0.094	0.099	--	0.094	0.099
	2000-2050 decadal trends in wind speed (m s^{-1})					
**FY**	0.036	--	0.042	0.052	0.039	0.063
**DJF**	0.047	--	0.078	0.091	--	0.12
**MAM**	0.052	--	--	0.067	0.06	0.065
**JJA**	--	--	--	--	--	--
**SON**	--	--	--	--	--	--
	2050-2100 decadal trends in wind speed (m s^{-1})					
**FY**	--	--	--	0.072	0.062	0.063
**DJF**	--	-0.077	--	0.088	0.076	0.093
**MAM**	--	--	--	0.071	0.035	0.08
**JJA**	--	--	-0.041	0.078	--	--
**SON**	--	--	--	--	0.101	--

table S1. Decadal trends in wind speed (m s^{-1}) for the six scenarios shown in Fig. 1, for each 50-year period studied. Only statistically significant ($p < 0.05$) trends are shown.

table S2

	90°S : 50°S	90°S : 70°S	70°S : 50°S	50°S : 30°S
**full year** (m s^{-1})	-0.036	-0.548	0.009	0.467
**DJF** (m s^{-1})	-0.1	-0.866	-0.033	0.425
**MAM** (m s^{-1})	-0.016	-0.654	0.04	-0.436
**JJA** (m s^{-1})	0.012	-0.302	0.039	1.361
**SON** (m s^{-1})	-0.04	-0.37	-0.011	0.518

table S2. Over-water wind speed bias (m s^{-1}) of the UKESM1 climatology (1940-2020) relative to the ERA5 reanalysis product, subdivided by latitude band.

table S3

	1940-2020 decadal trends in wind speed (m s^{-1})		1940-1980 decadal trends in wind speed (m s^{-1})		1980-2020 decadal trends in wind speed (m s^{-1})	
	ERA5	UKESM 1	ERA5	UKESM 1	ERA5	UKESM 1
**FY**	0.085	0.066	--	0.044	0.062	0.109
**DJF**	0.136	0.128	--	--	0.121	0.203
**MAM**	0.043	0.036	-0.099	--	--	0.099
**JJA**	0.084	0.026	--	--	0.065	0.1
**SON**	0.077	0.075	--	--	--	--

table S3. Decadal trends in mean UKESM1 and ERA5 winds (m s^{-1}) for the historical time period, south of 50°S. Only statistically significant ($p < 0.05$) trends are shown.

table S4

	1940-2020 mean jet position (° latitude)		1940-2020 Decadal trend in jet position (° latitude)		1940-1980 Decadal trend in jet position (° latitude)		1980-2020 Decadal trend in jet position (° latitude)	
full year	ERA5	UKESM1	ERA5	UKESM1	ERA5	UKESM1	ERA5	UKESM1
**DJF**	-51.52	-51.26	-0.195	-0.362	--	--	-0.3	-0.624
**MAM**	-54.16	-53.00	0.121	--	--	--	--	--
**JJA**	-53.08	-50.73	0.15	--	--	--	--	-0.542
**SON**	-53.61	-51.95	--	-0.186	--	--	--	--

table S4. Mean jet position and decadal trends (° latitude) in the position of maximum wind speed (wind jet). Only statistically significant trends at $p < 0.05$ shown.

table S5

		1950-2000		2000-2050		2050-2100	
scenario	quantity	ODS-driven	GHG-driven	ODS-driven	GHG-driven	ODS-driven	GHG-driven
SSP1	WIND (m s^{-1})	0.37	--	--	0.18	--	--
SSP3	WIND (m s^{-1})	0.37	--	--	0.26	--	0.36
SSP1	SST (°C)	-0.18	--	0.42	0.49	--	--
SSP3	SST (°C)	-0.18	--	0.34	0.59	--	1.56
SSP1	MLD (m)	10.7	-3.2	-5.1	-2.6	--	--
SSP3	MLD (m)	10.7	-3.2	-4.7	-2.6	--	-4.9
SSP1	MOC (Sv)	4.4	--	--	2.2	--	--
SSP3	MOC (Sv)	4.4	--	--	3.8	--	3.8

table S5. Contribution of ODS (blue bars) and GHG emissions (grey bars) to 50-year changes in mean overwater wind speed, SST, MLD, and MOC estimated from linear trends (see also Fig. 3). Only changes from significant trends ($p < 0.05$) are shown.

Table S6

		1950-2000		2000-2050		2050-2100	
		SSP1	SSP3	SSP1	SSP3	SSP1	SSP3
**Estimates from observationally-constrained biogeochemical data**							
**biology**	**data source**						
iron dFe/dz (nmol $^{-1}$ m^{-1})	GEOTRACES IDP2021	0.00053	0.00053	0.00053	0.00053	0.00053	0.00053
iron $dFe_{deep-surface}$ (nmol L^{-1})	GEOTRACES IDP2021	0.226	0.226	0.226	0.226	0.226	0.226
diatoms (µmol C L^{-1})	MAREDAT 2016	1.6	1.6	1.6	1.6	1.6	1.6
**carbonate chemistry**	**data source**						
DIC (µmol L^{-1})	GLODAPv2	2170	2170	2209	2214	2224	2275
TA (µmol L^{-1})	GLODAPv2	2350	2350	2350	2350	2350	2350
surf. oc. pCO2 (µatm)	calculated	288	288	378	393	421	642
dDIC/dz (µmol L^{-1} m^{-1})	GLODAPv2	0.41	0.41	0.35	0.34	0.33	0.25
$dDIC_{deep-surface}$ (µmol L^{-1})	GLODAPv2	143.46	143.46	115.79	112.19	105.58	69.21
**Values from the UKESM1 MEDUSA model**							
**biology**							
iron dFe/dz (nmol L^{-1} m^{-1})		0.00054	0.00054	0.00054	0.00055	0.00062	0.0007
iron $dFe_{deep-surface}$ (nmol L^{-1})		0.055	0.055	0.058	0.058	0.051	0.052
diatoms (µmol C L^{-1})		0.14	0.14	0.13	0.13	0.14	0.15
**carbonate chemistry**							
DIC (µmol L^{-1})		2189	2189	2220	2223	2226	2269
TA (µmol L^{-1})		2355	2355	2357	2357	2348	2346
surf. oc. pCO2 (µatm)		315	315	391	402	437	634
dDIC/dz (µmol L^{-1} m^{-1})		0.22	0.22	0.17	0.16	0.19	0.11
$dDIC_{deep-surface}$ (µmol L^{-1})		96.5	96.5	68.58	65.17	65.92	25.48

table S6. Estimates of biogeochemical values constrained by present-day observations and produced by the UKESM1 MEDUSA model, averaged over 50-year time periods for the two SSPs. For the method used for estimating future values for the biogeochemical parameters

based on present-day datasets, see Methods, sections “Observationally-constrained datasets – carbonate chemistry” and “Assumptions when using observationally-constrained values”

table S7

known biases in UKESM1 MEDUSA biogeochemical model	
**bias**	**likely effect on change in surface DIC**
reduced surface DIC vertical gradient	dampens changes in surface DIC due to changes in circulation
low diatom biomass	dampens changes in surface DIC due to changes in biological activity
low iron vertical gradient	dampens changes in surface DIC due to changes in biological activity
assumptions when using biologically-constrained biogeochemical values to estimate future DIC values	
**assumption**	**likely effect on change in surface DIC**
Total alkalinity (TA) is constant	Overestimates changes in surface DIC relative to a case with likely future decrease of TA, which would decrease DIC uptake efficiency (see text)
Biomass is constant and only responds to changes in iron	Could change in either direction, affecting ΔpCO_2, but historical calculated effects are small, so differences are likely to be small
ΔpCO_2 is constant	Multiple processes can alter ΔpCO_2 (see text), but effects are likely to be secondary compared to change in surface DIC due to anthropogenic changes in atmospheric CO_2
Vertical distribution of anthropogenic DIC is constant	Could change in either direction due to circulation, but effects are likely small (see sensitivity test in fig. S6)

table S7: A summary of known biases in the UKESM1 MEDUSA model and assumptions made when estimating future surface DIC from present-day observations.
